# Supplementary material for: Clinical Skills Tutoring Program (CSTP): Developing a Curriculum for Medical Student Clinical Skills Peer Tutors
Source: MedEdPORTAL. 2022 Feb 14;18:11225. doi: 10.15766/mep_2374-8265.11225 (PMC8841391; doi:10.15766/mep_2374-8265.11225)
Supplement: Supplementary file 1 — Tutor Curriculum Learning Objectives and Content.docxTutor Curriculum Supplement.docxTutor Curriculum Nuts and Bolts.docxTutor Checklist.docxCSTP Facilitator Guide for Tutor Training Session.docxTutor Training Session Survey.docxTutor Participant Survey.docxStudent Participant Survey.docx [file mep_2374-8265.11225-s001.zip › A. Tutor Curriculum Learning Objectives and Content.docx]

**Clinical Skills Tutoring Program (CSTP)**

**Tutor Curriculum**

*Welcome to the Clinical Skills Tutoring Program! We are eager to work with you and hope you have a wonderful experience in this program. Our overall goal and objectives for this tutor curriculum are as follows:*

**PROGRAM GOAL:**

To recruit, train, and support clinical skill peer tutors that in turn serve other students to gain knowledge, skills, attitudes, and confidence in clinical skills.

**LEARNING OBJECTIVES:**

By the end of your participation as a peer tutor in the CSTP, you will be able to:

1. Define learning theories and processes that support peer tutoring, including cognitive congruence, social congruence, self-regulated learning, and the master adaptive learner conceptual model.
2. Analyze and discuss prior performance reports and recorded videos related to clinical skills with a student.
3. Describe the components of an individualized competency-based learning plan (ILP), formulate an ILP with the student, design a customized schedule, and implement a coaching plan with the student to achieve the goals of the ILP.
4. Deliver effective feedback on self-efficacy, goal setting, strategic planning, self-monitoring, self-evaluation, attribution beliefs, and adaptive changes to a student based on direct observations of clinical skills performance.
5. Model proficiency in hypothesis-driven history taking and physical exam skills, diagnostics/clinical reasoning, and communication skills relevant to commonly encountered symptoms and diagnoses.
6. Display effective skills in communication and coordination with the CSTP program directors and Bridges coaches to provide coordinated support to the student.

**Learning Objective 1:** Define learning theories and processes that support peer tutoring, including cognitive congruence, social congruence, self-regulated learning, and the master adaptive learner conceptual model.

Peer tutoring has a long-standing history in medical education. The benefits of peer-tutoring have been well-studied. Studies describe cognitive and social congruence as the root of these benefits as they help shape the tutor-student relationship. Thus, understanding the definitions and strategies of cognitive and social congruence and how to use these theories to build and frame a connection with your student will be critical to your success as a tutor.

**Cognitive congruence** is defined broadly as tutors and students sharing the same knowledge framework. One example of this is tutors being able to understand where students are struggling and how to overcome those challenges, based on prior similar learning difficulties. Another example is being able to organize topics for the student based on relative clinical importance (based on experience gained from the wards) and difficulty (based on experience gained from clinical exam preparation).

An important component of cognitive congruence is the ability for student tutors to use language that is familiar to students, often harnessing the innate ability to explain difficult concepts at an appropriate level. For example, students gravitate towards instructors that communicate an initially difficult topic in an easy-to-understand fashion that is both interesting and thought-provoking.^2^ Students find it valuable when their tutors acknowledge 1) reasons for why the content was confusing for the tutor when they learned it and 2) how they made sense of the material when learning it for the first time.^1^

**Social congruence** is defined as tutors and students sharing similar social roles in the education hierarchy. This creates a safe learning environment for students as they perceive their peer tutors as being more supportive and empathic towards their learning needs. In addition, social congruence removes learning barriers often referred to as “the hidden curriculum”, where learner expectation and goals are not clearly communicated or established due to components of the medical hierarchy. Several studies indicate that students are more engaged in class with peer tutors through openly receiving feedback, taking risks, and asking questions. Through social congruence, tutoring sessions can mirror case-based learning in that a tutor is a facilitator, using probing questions to maintain student focus, provide clarification, answer questions, and bring closure to the discussion.^2^ Please view specific strategies and examples of cognitive and social congruence located in **Appendix B.**

Cognitive and social congruence will be used to promote self-regulated learning. You will help your students build the skills to identify and change their approach to learning in order to achieve mastery of a given task (e.g. clinical skills). Details on how to accomplish this are outlined in **Learning Objective 4.**

In the clinical environment, there is improvement to performance with feedback and repetition that we see with self-regulated learning. It is equally important to encourage problem-solving skills, where the student reframes the problem in a way that allows them to explore new concepts and invent new solutions. This is a skill called adaptive expertise that is acquired by students under the Master Adaptive Learner model. In this model, tutors will assist students in four steps: 1) planning, 2) learning, 3) assessing, and 4) adjusting. During the planning phase, you will help your student identify gaps in knowledge and describe learning strategies and resources to help resolve those gaps. Next, you will help your student learn by critically evaluating which strategies and resources are effective or ineffective at achieving student goals. Afterwards, students will be assessed on their knowledge gained through practice, observation, and feedback. Lastly, the adjustment phase will solidify the student’s new skillset into daily practice, identify any remaining gaps, and create new learning goals, if needed.

**Key Reading for Objective 1:**

1. Lockspeiser TM, O’Sullivan P, Teherani A, Muller J.Understanding the experience of being taught by peers: The value of social and cognitive congruence. *Advances in Health Sciences Education*, 2008;13(3):361-372. <https://doi.org/10.1007/s10459-006-9049-8>
2. Loda T, Erschens R, Loenneker H, et al. Cognitive and social congruence in peer-assisted learning – A scoping review. *PLOS ONE.* 2019;14(9):e0222224. <https://doi.org/10.1371/journal.pone.0222224>
3. Watson SL, Koehler AA, Ertmer P, Kim W, Rico R. An Expert Instructor’s Use of Social Congruence, Cognitive Congruence, and Expertise in an Online Case-Based Instructional Design Course. *Interdisciplinary Journal of Problem-Based Learning.* 2017;12(1):12. <https://doi.org/10.7771/1541-5015.1633>
4. Cutrer WB, Miller B., Pusic MV, et al. Fostering the Development of Master Adaptive Learners: A Conceptual Model to Guide Skill Acquisition in Medical Education. *Acad Med*. 2017;92(1):70-75. <https://doi.org/10.1097/ACM.0000000000001323>

**Evaluate your understanding with the following:**

Imagine a scenario where the student is having a challenging time receiving feedback related to the cardiac exam. How would you use cognitive and social congruence strategies to improve their cardiac exam skills?

**Learning Objective 2:** Analyze and discuss prior performance reports and recorded videos related to clinical skills with a student.

During the first meeting with the student, you may review prior score reports and selected performance videos with your student. Reports and videos may be available for DOCS 1/2/3, mini CPX, CPX, Clerkships, and Step 2 CS. The CSTP program directors will review reports and videos and share key features with the tutor and student pair. Sharing these actual reports and videos with the tutor is optional for the student (you will not have access to the student’s dashboard); invite them to do so as it may enhance the discussion between the tutor and the student, but emphasize it is optional. **.** You may find it helpful to ask your student to allow you to view selected materials/videos/score reports in advance of the first meeting to allow for more preparation time.

In order to make this a psychologically safe learning experience for the student, the discussion about prior reports and videos should take place in a private one-on-one environment on the UCSF campus (e.g., private library classrooms, medical education building, nursing building). Explicitly state that clinical skills performance reports are a private part of a medical student’s experience. It is important to emphasize that everything you see and discuss with the student related to their performance reports will be kept confidential.

Below is guidance on how to approach these materials with your student:

**Task #1: Review prior clinical skills performance reports and clerkship performance reports**

1. **What are the student’s relative strengths?**

- Assess whether there are sections that they scored at or higher than the mean.
- Ask the student what they perceive their areas of strength are. This allows you to tailor your curriculum around those strengths so that you don’t over-emphasize material the student is already comfortable with.

1. **What are the student’s areas for growth?**

- Assess whether there are sections that they consistently score below the mean.
- Ask the student what they believe their areas for growth are or where they struggle
- Ask the student if they have any learning goals already set for themselves prior to the session.

View example scenarios illustrating Task #1 in **Appendix B**.

**Task #2. Reviewing standardized patient encounter videos**

When reviewing the standardized patient encounter videos, welcome your student into an open discussion. Before you start, preface this by stating, for example, the following:

*“While the video is playing, I may pause at certain moments to discuss with you what I am observing. I would love to hear your input about what you are seeing and thinking in that moment, too. While the video is playing, I may also talk about things that I am watching for and you can stop me and ask me questions at any time. How does that sound? You can also pause the video at any time to ask questions/make observations.”*

Consider the below while watching the video:

1. Divide the encounter into three sections: beginning, middle, and end.
2. In each section, what was done correctly or incorrectly? (frequently pause to discuss both things that are going well and things that are not with the student)
3. If the encounter went well, what caused it to go well?
4. If the encounter did not go well, what were the major concerns?
5. What learning goals can we create to address these gaps? How can I help the student identify these areas for growth and verbalize them into a learning objective?
6. While giving feedback, how is my student feeling? (receptive, embarrassed, sad, frustrated, etc.) What can you do to address the emotional/psychological state of the student?
7. How can I make my student feel hopeful about future improvements?

**Task #3. Develop a cognitive framework that works for you and your student**

Since there are a lot of elements occurring during a patient encounter video, it makes sense to use a cognitive framework to standardize your approach to reviewing them with your student. We share one such framework in **Appendix B.** You may adopt this framework if it works well for you; also feel free to adopt a different framework if more appropriate for your own teaching style and/or student’s learning style. The purpose of the sample framework is to jumpstart ideas in your head on how to discuss and walk through a clinical encounter with your student. Additional examples of frameworks commonly used are found in textbook resources such as First Aid for Step 2 CS and The Patient History: Evidence-Based Approach.

Please see **Appendix B** for a sample framework.

**Task #4. Deliver Targeted Feedback**

We will cover strategies and best practices in giving feedback for self-regulated learners in **Learning Objective 4.**

**Evaluate your understanding with the following:**

You are about to meet with your student for the first time to review their recent CPX report. Unfortunately, they did not pass the exam. Where will you meet, how will you go about reviewing the report together? Will you invite the student to watch their CPX videos together with you? If so, which ones will you watch and what is one framework you can use to review the videos together?

**Learning Objective 3:** Describe the components of an individualized competency-based learning plan (ILP), formulate an ILP with the student, design a customized schedule, and implement a coaching plan with the student to achieve the goals of the ILP.

You are expected to review and discuss the student’s Individualized Learning Plan (ILP) during the start and end of every tutoring session, and you will email the ILP to the CSTP co-directors monthly (or at least every 4 sessions). An ILP is created by the student and is made up of different goals, plans to achieve those goals, and outcome measures to determine whether the goal has been met. The purpose of the ILP is to help you and your student track learning goals over time and see how they evolve. In addition, this process helps provide reinforcing feedback to students on achieving their goals and setting new goals. See **Appendix B** for the ILP template that students will receive.

As part of the ILP, you are expected to create “SMART” (**S**pecific, **M**easurable, **A**ttainable, **R**elevant, and **T**imebound) goals with your student.

An example of a SMART goal may be: “At the end of this tutoring program, I aim to share with all standardized patients during encounters what my top diagnosis is and what the key next steps are for the plan.”

Below is a guide (adapted from Lawlor, 2012, and Lockspeiser and Kaul, 2016), for tutors working with students when creating and refining the ILP and SMART goals (citation: author-owned):


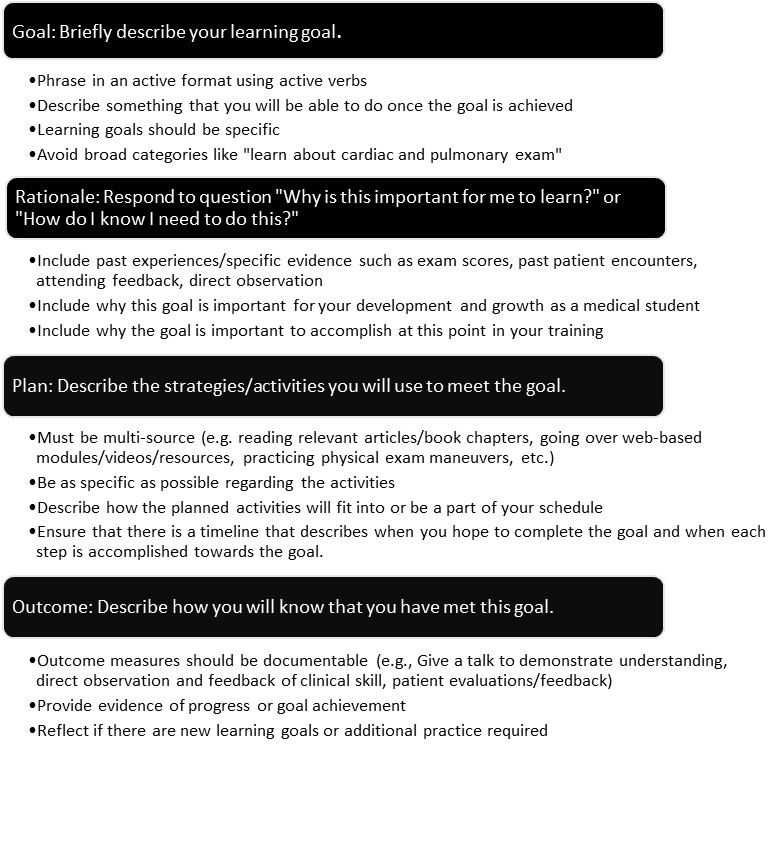


**Key Reading for Objective 3:**

1. Lawlor KB. **Smart goals: How the application of smart goals can contribute to achievement of student learning outcomes**. *Developments in Business Simulation and Experiential Learning*. 2012;39.
2. Lockspeiser TM, Kaul P. Using Individualized Learning Plans to Facilitate Learner-Centered Teaching. *Journal of Pediatric and Adolescent Gynecology*. 2016;29:214–217. <https://doi.org/10.1016/j.jpag.2015.10.020>

**Evaluate your understanding with the following:**

You are meeting your student for the third time. Over the last three sessions you have reviewed their ILP prior to and after each session. You feel like the student is not meeting the goals on their ILP. You note that some of the goals are not formatted in the “SMART” framework. What strategies would you use to communicate your concern about their goals and how may you adjust the goals so that they are achievable during the tutoring sessions?

**Learning Objective 4:** Deliver effective feedback on self-efficacy, goal setting, strategic planning, self-monitoring, self-evaluation, attribution beliefs, and adaptive changes to a student based on direct observations of clinical skills performance.

Self-regulated learning is defined as a three-phase cyclical process where individuals (students) proactively plan, self-monitor, and adapt their actions to achieve their goals. The three phases are labeled as 1) forethought, 2) performance, and 3) self-evaluation. Forethought is the planning phase before any actions are performed. This involves goal setting and planning specific steps towards reaching that goal. This phase is followed by performance which are the actions performed to execute the planned strategy. The third phase, self-evaluation, is used to reflect on the actions performed and make any changes to your strategy to ensure that the initial goal is achieved. Feedback is critical to keep this three-phase cycle running smoothly. A useful diagram adapted from Sandars and Cleary, 2011, that provides further details on self-regulated learning and the components of each phase can be found in **Appendix B.**

Based on the model proposed by Leggett *et al.,* 2019, feedback to students should be given on the following key self-regulated learning processes based on direct observation of clinical skills practice. The topics and their definitions are as follows:

| **Key Process** | **Definition** |
| --- | --- |
| Self-Efficacy | The extent to which an individual feels confident about whether they can achieve a task |
| Goal-Setting | Steps towards achieving the desired goal, should be specific and measurable |
| Strategic Planning | Specific strategies or techniques (such as memorization of procedures) to help learners monitor and regulate their progression through the task |
| Self-monitoring | Adapting and changing their strategies by constantly checking-in on their progress towards achieving their desired goals |
| Self-evaluation | Critical reflection and incorporation of feedback through writing or question-asking to improve future performance |
| Attribution beliefs | Learner’s specific beliefs about the causes of their performance (e.g., ability to identify and acknowledge own mistakes instead of blaming factors outside of their control) |
| Adaptive changes | Learner’s ability to adjust their approach during real time tasks in order to improve their performance. Best utilized after a poor performance. |

The following table provides an overview of each key process and how to approach discussions with students and giving feedback in each area. More detailed explanations are provided by the reference articles listed following this section.

| **Key Process** | **Example Questions to Assess Student** | **Goal of Feedback** | **Example of Feedback** |
| --- | --- | --- | --- |
| Self-efficacy (Pre) | “How confident are you in doing…” | Identify baseline confidence to compare after intervention | “Let’s come up with a plan to increase your confidence moving forward.” |
| Goal setting | “What learning goals do you have in mind to help you…” | Provide feedback to ensure goals are SMART | “Let’s discuss ways to make this goal more specific and measurable.”  “Let’s set a date when you want to accomplish this next step.” |
| Strategic planning | “What do you think are the next steps needed to accomplish…” | Encourage student to create strategies and organize a plan for their goals | “I noticed you might be skipping a few steps towards goal A. Let’s discuss how we can fill in those gaps.” |
| Self-monitoring | “What are your thoughts about what you tried?” | Encourage student to think about their progress | “You mentioned to me that you felt hesitant, because you weren’t sure if your strategy would be effective in this scenario. Let’s discuss how we can adjust your strategy for future cases.” |
| Self-evaluation | “What happened in that case that made you feel that it was successful/unsuccessful?” | Prompt students to think about what outcomes they desire for their goals | “Do you think you have performed well thus far or have you made any mistakes?” |
| Attribution beliefs | “Why do you think that you could not achieve…” | Encourage students to reflect on their own efforts, abilities, and mistakes | “Based on what you told me, I would recommend thinking about how to improve the ability to…” |
| Adaptive changes | “What do you need to do to become successful on your next attempt?” | Have student think about changing their strategy | “What would you have done differently if you had to try again? Why would you choose to do that differently?” |
| Self-efficacy (Post) | “How confident do you feel now in…” | Identify post-intervention confidence to compare | “Based on your self-rating now, you seem to have made improvements in these areas. In addition, we created some new learning goals such as ...” |

The diagram below is designed to help you visualize at what stages feedback should be given for each topic.

**Key Reading for Objective 4:**

1. Leggett H, Sandars J, Roberts T. Twelve tips on how to provide self-regulated learning (SRL) enhanced feedback on clinical performance. *Med Teach.* 2019:41(2):147–151. <https://doi.org/10.1080/0142159X.2017.1407868>
2. Sandars J, Cleary TJ. Self-regulation theory: Applications to medical education: AMEE Guide No. 58. *Med Teach*. 2011;33(11):875-886. <https://doi.org/10.3109/0142159X.2011.595434>

**Evaluate your understanding with the following:**

You and your student have identified taking a focused history for abdominal pain as a general learning goal. Describe how you would engage your student in discussions about self-efficacy. Provide an example of a SMART goal you would create with your student. How would you walk your student through strategic planning, self-monitoring, and self-evaluation? How would you provide feedback on attribution beliefs and adaptive changes if your student doesn’t achieve their initial goals?

**Learning Objective 5:** Model proficiency in hypothesis-driven history taking and physical exam skills, diagnostics/clinical reasoning, and communication skills relevant to commonly encountered symptoms and diagnoses.

This tutor curriculum does not specifically cover clinical skills content as you are expected to have gained core competence through first and second-year preclinical skills training as well as your third and fourth-year clinical rotations and electives. Material will be provided on the CLE page for review, including history taking, physical exam skills, diagnostic/clinical reasoning, and communication skills relevant to commonly encountered symptoms and diagnoses. If you feel uncomfortable about tutoring in these areas, please discuss with the program directors. The program directors are a resource to you. The goal of the CSTP is for you to model proficiency and continue to improve your own clinical skills as you work with other students.

The following additional resources may be beneficial in your review of these clinical skills topics. This list is not meant to be comprehensive, but are common resources used by UCSF students.

Resources in the second column are textbooks that are available in the UCSF library. Resources in the third column are web-based, free-to-access for UCSF students.

| **Content Area** | **Textbooks** | **Web-Based** |
| --- | --- | --- |
| **History-Taking** | - The Patient History: Evidence-Based Approach - First Aid for Step 2 CS | - Bate’s Clinical Cases - UCSD’s Practical Guide to Clinical Medicine |
| **Physical Exam** |  | - Bate’s Physical Exam Videos - UCSF Physical Exam Checklist - UCSF MSK and Neuro Exam Apps - UCSD’s Practical Guide to Clinical Medicine |
| **Diagnostic/Clinical Reasoning** |  | - OnlineMedEd - Bate’s OSCE Clinical Skills Videos - Aquifer Family Medicine Cases (for counseling and screening guidelines) |
| **Patient Communication** |  | - Bate’s OSCE Clinical Skills Videos |
| **Note Writing** |  | - UCSD’s Practical Guideline for Clinical Medicine - NBME Step 2 CS Note Writing Practice |

**Learning Objective 6:** Display effective skills in communication and coordination with the CSTP program directors and Bridges coaches to provide coordinated support to the student.

You will arrange meetings with the student’s coach and the clinical skills peer tutoring program directors prior to the start of the tutoring sessions. Meetings can be arranged for either in-person or over the phone. The goals of these meetings are to review prior performance, organize areas for improvement for the student, and discuss any specific needs or preferences the student may have in learning and receiving feedback. In addition, you are asked to arrange a check-in meeting with the program directors within your first two sessions with the student. The goal of this meeting is to check-in about progress with the student and discuss any difficulties with logistics (scheduling) or content. These meetings will provide an opportunity for you to ask additional questions and provide valuable feedback, while ensuring a positive relationship with your student. The student should be made aware that these meetings are taking place as part of the CSTP and that all discussions related to the student are absolutely confidential.

You can check-in with program directors and the student’s coach after any session if you have questions or concerns. You will be assigned a primary program director as a point of contact throughout your involvement in the program.

The student’s coach is a valuable resource during all phases of the tutor-student relationship, because the coach has the longest and most significant longitudinal relationship with the student during all four years of medical school. They are the primary observers of clinical skills during pre-clinical years and are your student’s go-to resource for questions and feedback. When working with the student’s coach, consider the following areas of discussion: 1) eliciting input from the coach and 2) sharing observations and feedback with the coach.

**Eliciting input from the coach:**

*Note: For some groups, it may be more appropriate for the student-tutor pair to discuss with coach together rather than a tutor-coach 1-to-1 discussion.*

1. Beginning of tutoring:

- What are the student’s strengths and areas for improvement?
- Have these areas been discussed before and in what way?
- In what major areas do you feel I can benefit the student?
- What is the student’s learning style like? (visual, hands-on, written)
- How does the student prefer receiving feedback?
- Anything else I should know about the student to help?

1. During tutoring:

- Sharing progress of student during tutoring sessions
- Asking for additional advice on giving feedback to student
- Organizing a meeting if there is conflict regarding communication and learning style preference

1. End of tutoring:

- Sharing observations and feedback with the coach
- Discussing future goals or plans for more sessions

**Sharing observations and feedback with the coach and/or program directors:**

At the conclusion of the tutor-student relationship, you will be responsible for checking in with the student’s coach and the program directors to provide a final update on your student’s progress, achievements, and future learning goals. Because the discussion will contain private information regarding student performance, the best practice is for the meeting to take place with the student-tutor pair and the coach. This ensures transparency between each member of the team and promotes a safe and supportive environment for the student.

In anticipation for this, it may be beneficial for you to keep your old notes taken during clinical observation sessions with your student as well as records of the student’s ILP and learning goals during each session. That way your observations and feedback given to the coach can be not only specific but paint an accurate representation of your student through several clinical examples. You may condense these materials into a single document for the meeting and preparation time is expected to be no more than 10-15 minutes. Preparation of these materials as well as meeting time will be factored into the tutor salary.

Examples of things to discuss during this meeting include, but are not limited to:

1. Student ILP and Learning Goals – their achievements and future goals
2. Specific areas categorized as strengths
3. Specific areas categorized as areas needing improving – discuss progress and milestones
4. Student’s current clinical skills performance compared to prior
5. Student’s future learning goals
6. Discussing how the coach can help implement future learning goals and reinforce student’s progress

Documents that would be helpful to keep for this discussion:

1. Student ILP documents
2. Student’s SMART learning goals
3. Notes on student strength and areas for improvement
4. Teaching materials that you covered with the student
5. Notes on student’s clinical performance on standardized patient cases

**Evaluate your understanding with the following:**

You just completed your final session with the student. You think things went well and the student met several important goals on their ILP. How will you go about summarizing your experience with the CSTP program directors and the student’s coach? How will you organize next steps (if any) for these faculty members?
