# Supplementary material for: Clinical Skills Tutoring Program (CSTP): Developing a Curriculum for Medical Student Clinical Skills Peer Tutors
Source: MedEdPORTAL. 2022 Feb 14;18:11225. doi: 10.15766/mep_2374-8265.11225 (PMC8841391; doi:10.15766/mep_2374-8265.11225)
Supplement: Supplementary file 1 — Tutor Curriculum Learning Objectives and Content.docxTutor Curriculum Supplement.docxTutor Curriculum Nuts and Bolts.docxTutor Checklist.docxCSTP Facilitator Guide for Tutor Training Session.docxTutor Training Session Survey.docxTutor Participant Survey.docxStudent Participant Survey.docx [file mep_2374-8265.11225-s001.zip › B. Tutor Curriculum Supplement.docx]

**Clinical Skills Tutoring Program (CSTP)**

**Tutor Curriculum Appendix**

*This document provides supplementary material for the learning objectives and content in the tutor curriculum. Contents in this document are referenced in the tutor curriculum.*

***Note: To navigate this document, Cntrl+Left Click on the blue hyperlinks will take you to the corresponding section.***

**Table of Contents**

[Learning Objective 1:](#LearningObjective1) Define learning theories and processes that support peer tutoring, including cognitive congruence, social congruence, self-regulated learning, and the master adaptive learner conceptual model.

[Learning Objective 2:](#LearningObjective2) Analyze and discuss prior performance reports and recorded videos related to clinical skills with a student.

- [Example Scenarios on Reviewing Clinical Skills Performance Reports](#ExampleScenarios)
- [A Cognitive Framework for Standardized Patient Encounters](#CognitiveFramework)

[Learning Objective 3:](#LearningObjective3) Describe the components of an individualized competency-based learning plan (ILP), formulate an ILP with the student, design a customized schedule, and implement a coaching plan with the student to achieve the goals of the ILP.

[Learning Objective 4:](#LearningObjective4) Deliver effective feedback on self-efficacy, goal setting, strategic planning, self-monitoring, self-evaluation, attribution beliefs, and adaptive changes to a student based on direct observations of clinical skills performance.

**Learning Objective 1:** Define learning theories and processes that support peer tutoring, including cognitive congruence, social congruence, self-regulated learning, and the master adaptive learner conceptual model.

***Table 1.*** *Five Common Cognitive Congruence Strategies with Examples (adapted from Watson et al., 2017)*

| Cognitive congruence Strategy | Example |
| --- | --- |
| 1. emphasizing important ideas | “Michael, I notice that you made significant improvements in your ability to build rapport with the patient. Let’s discuss some of the strategies you have incorporated into your patient visit while we watch the encounter video together.” |
| 1. clarifies idea or discussion | “Before I move on, Anne, do you have any questions for me? This was a difficult and confusing topic for many students when we first learned this, so it’s completely normal to review this further.” |
| 1. direct student attention | “The next step in our discussion leads us to testing range of motion in the upper extremities. Would you like to walk me through what you have learned so far?” |
| 1. provide example | “This reminds me of a patient encounter I had during my third-year rotations that illustrates the importance of nutritional counseling in diabetes management. Would you like to go through this example patient case together?” |
| 1. Provide summary | “At the end of this case, the key learning points were…”  “After discussing this patient encounter, your key strengths were … “  “After watching this patient encounter together, would you like to summarize for me the learning goals we have created?” |

***Table 2.*** *Five Common Social Congruence Strategies with Examples (adapted from Watson et al., 2017)*

| Social Congruence Strategy | Example |
| --- | --- |
| 1. Acknowledging student ideas | “That’s an excellent learning plan, Tony! I particularly appreciate the attention to detail in dividing the neuro exam into separate components to practice. This makes the learning plan more specific and actionable.” |
| 1. Inviting students to join and continue the discussion | “When thinking through our differential for chest pain, we did a thorough job covering issues involving the heart. What other organ systems should we consider?” |
| 1. Using students’ names while conversing | “Gary and Anne, you both did a wonderful job demonstrating the cardiac exam. Would you mind sharing with the group your strategy for approaching this exam?” |
| 1. Showing enthusiasm about discussion topics | “Mary, you did an excellent job asking questions to clarify key concepts today. Keep this up in our future sessions, great work!” |
| 1. Expressing approval of students’ ideas | “Anne, I think it’s a wonderful idea to talk about your concern about time management in the patient encounter. Please tell me what has been bothering you the most?” |

**References:**

1. Watson SL, Koehler AA, Ertmer P, Kim W, Rico R. An Expert Instructor’s Use of Social Congruence, Cognitive Congruence, and Expertise in an Online Case-Based Instructional Design Course. *Interdisciplinary Journal of Problem-Based Learning*. 2017;12(1):12. <https://doi.org/10.7771/1541-5015.1633>

**Learning Objective 2:** Analyze and discuss prior performance reports and recorded videos related to clinical skills with a student.

**Example scenarios on reviewing clinical skills performance reports.**

**Example scenario 1:**

**Tutor:** “*On the case with Mr. Johnson, it looks like your history-taking portion was scored very highly, do you perceive that as a strength?”*

**Student:** *“Sort of. I feel comfortable gathering all the relevant information from my patients, but when I’m nervous, I do sometimes go all over the place, asking their medications and allergies and then going back to the history and then going to social history and losing my place. Sometimes I ask extra history questions during the physical exam as well.”*

**Tutor:** “*It seems like you have some thoughts about a good learning goal for yourself. Let’s make a learning plan to streamline history-taking so that it becomes more organized, even when you’re nervous. What do you think about that?”*

*Scenario Discussion:* The teaching skills used in this scenario include asking open-ended questions assessing a student’s strengths. By asking broad questions, the student is given the opportunity to assess global strengths and oftentimes, bring up perceived weaknesses while thinking about their strengths. Whenever a student addresses a potential area for growth, approach them with the idea of creating a learning goal and addressing it during current or future sessions.

**Example scenario 2:**

**Tutor:** “*As we look over your performance report, could you tell me what areas of clinical skills you would like to work on the most?”***Student:** ”*There are several areas. I think I run out of time so I can’t do patient counseling, so time management is a big thing. Also, I always miss something on the physical exam that is important to perform. I think I also take too long on the history-taking process.”***Tutor:** “*That is great that you’ve been thinking deeply about these areas for growth. Let’s focus on one specific area, which would you like to explore first?”***Student:** “*Let’s work on reducing the time it takes for me to take a history”***Tutor:** “*Sounds like a great plan, please tell me more about how you normally approach history-taking. Can you start from the very beginning of a typical encounter so I can understand your approach? ”*

*Scenario Discussion:* In this scenario, the teaching skills used include starting with open-ended questions. If the student provides short responses after a series of open-ended questions, it may be helpful to ask more specific questions such as “How comfortable are you with history-taking?” or “Many people find taking a hypothesis-driven history in a set amount of time during standardized exams to be really hard. Do you ever have any difficulty with this during cases?”. Another technique highlighted in this scenario is addressing a single learning issue when the student brings up multiple (even if they may be related). It is a good approach to create a learning goal identifying a single, high priority learning issues as this makes the goal more specific, targeted, and trackable. If the problem is too broad, (Ex. I can’t finish an encounter in 15 minutes”) ask more details to narrow the learning objective.

**A Cognitive Framework for Standardized Patient Encounters**

**Cognitive Framework Example: Focusing on the “overview” and the “details” of the encounter**

This framework divides the patient encounter into two categories: “overview” and “details”. Overview is defined as the organizational aspects of the encounter, such as the structural and planning elements. Details is defined as the specifics of the encounter, such as specific elements of the history and physical exam. Approach both categories in a step-by-step fashion.

**Approaching the Overview (“Overall feel of the encounter”):**

1. **Introduction and Hand Hygiene**

- Did student say both their First and Last Name?
- Was hand sanitizer used upon entering the room?
- Was hand sanitizer used again before the physical exam?

1. **Time Management**

- How much time was spent on the history? Physical exam? Counseling?
- Did student accomplish all tasks in 15 minutes?
- Was there any section that appeared rushed or incomplete?

1. **Patient Comfort and Draping**

- Was there good rapport? If not, why?
- Did the patient feel comfortable during the physical exam?
- Was proper draping performed to minimize exposure? Gown tied back up?
- Did the patient feel rushed at any point of the encounter?
- Technical jargon use, interrupting the patient, patient misunderstanding?

1. **Student Comfort**

- Student posture and eye contact
- Did the student have good pacing during the encounter?
- Did the student have good organization?
- Was the student familiar with where the instruments are located?
- Did the student use those instruments with proper technique?
- How did the student deliver bad news or provide reassurance?
- How comfortable was the student in the delivery of a plan?

**Approaching the Details (“Specifics of the Encounter”):**

1. **History of Present Illness**

- Did the student start off with an open-ended question? (e.g. what brings you in to see us today? Do you have any concerns today?)
- Did the student cover the OPQRST (onset, palliation, quality, radiation, severity, time) or OLD CARTS (onset, location/radiation, duration, character, aggravating factors, relieving factors, timing, severity) related to the patient’s chief complaint?
- Did the student ask more specific questions targeting differential diagnosis?

**-**What diagnoses were the student thinking of during the encounter?

- Did the student perform a pertinent review of systems?
- Was there a summary of the story provided back to the patient?

1. **Additional Medical History**

- Did the student ask for past medical history?
- Did the student ask for current medications, vitamins, and supplements?
- Did the student perform a brief medication reconciliation (dosage/how often taking)
- Did the student ask for patient allergies? Did they specify the reaction?
- Did the student assess family history?
- Did the student ask for social history (e.g. housing, occupation)
- Did the student ask for health-related behaviors (diet, exercise)
- Did the student ask questions for alcohol use, tobacco use, substance use and clarify amount and frequency?

1. **Physical Exam**

- Does the student explain to the patient what parts of the exam will be performed?
- Does the student talk the patient through what they are looking for?
- Does the student clarify if findings are “healthy and normal”?
- Are there any components of the exam that were missed?
- Are there any components of the exam that were not performed correctly?

1. **Closing the Encounter**

- Does the student summarize and explain the encounter in an organized fashion?
- Does the student clearly explain what might be going on and what tests are needed?
- Does the student clarify the reason behind ordering the tests and what the tests are?
- Does the student ask the patient for any additional questions?
- Does the student answer the questions in a professional manner?

**Learning Objective 3:** Describe the components of an individualized competency-based learning plan (ILP), formulate an ILP with the student, design a customized schedule, and implement a coaching plan with the student to achieve the goals of the ILP.

**Learning Goals Worksheet for Clinical Skills Peer Tutoring Program**

We are really looking forward to working with you in the Clinical Skills Tutoring Program. Before you begin your work with your peer tutor, we would like you to create two to three learning goals and start to think about how you will work on these goals with your peer tutor.

To come up with these goals and learning plans, we encourage you to think about the following:

- feedback you have received on clinical skills from your coach, preceptors, and attendings/residents in clinic and on the wards
- feedback you have received from standardized patients
- videos and score reports from clinical skills exams

You will then refine these learning goals with your peer tutor during your initial meeting(s).

To increase the usefulness of your goals, please make these goals “SMART” (**S**pecific, **M**easureable, **A**ttainable, **R**elevant and **T**imebound).

- An example “SMART” goal may be: “At the end of this tutoring program, I want to increase my comfort in taking a relevant social history in standardized patient encounters from my current 1/5 (where zero is not comfortable and five is the most comfortable) to 4/5.”

If you have any questions as you work on your goals, please feel free to email the co-directors of the Clinical Skills Peer Tutoring Program. Please email the goals sheet to the program co-directors when you have completed it.

Thank you.

☐ I **consent** to this data being part of **education research** related to the Clinical Skills Peer Tutoring Program

**Clinical Skills Learning Goal 1:**

**Feedback/data I have received related to this goal (e.g., what supports the decision to choose this goal):**

**Plan for how I will work on Learning Goal 1 with my tutor:**

**Clinical Skills Learning Goal 2:**

**Feedback/data I have received related to this goal (e.g., what supports the decision to choose this goal):**

**Plan for how I will work on Learning Goal 2 with my tutor:**

**Clinical Skills Learning Goal 3:**

**Feedback/data I have received related to this goal (e.g., what supports the decision to choose this goal):**

**Plan for how I will work on Learning Goal 3 with my tutor:**

**Learning Objective 4:** Deliver effective feedback on self-efficacy, goal setting, strategic planning, self-monitoring, self-evaluation, attribution beliefs, and adaptive changes to a student based on direct observations of clinical skills performance.

**Self-Regulated Learning Diagram**

**Figure 1.** Three phases of self-regulated learning. Figure adapted from Sanders and Cleary, 2011.

Performance phase:

- Do I have a clear understanding of the task?
- Does my learning goal make sense for the task being performed?
- Am I achieving my goals?
- Do I need to make changes?
- Do I need to modify my thoughts, emotions, or strategic plan?
- Do I need to modify the learning environment?

Forethought phase:

- What is the nature of the task?
- What is my learning goal?
- What kind of information, skills, and strategies do I need?
- How much time and resources will I need?
- What is my motivation?
- Do I need to modify the learning environment?

Self-reflection phase:

- Have I reached my goal?
- What worked? What resources or strategies were helpful?
- What did not work? What resources or strategies were ineffective?
- Would I do things differently next time?
- In what ways would I change my approach?
- Have I created any new follow-up learning goals?

**References:**

1. Leggett H, Sandars J, Roberts T. Twelve tips on how to provide self-regulated learning (SRL) enhanced feedback on clinical performance. *Med Teach*. 2019;41(2):147–151. https://doi.org/10.1080/0142159X.2017.1407868
2. Sandars J,Cleary TJ. Self-regulation theory: Applications to medical education: AMEE Guide No. 58. *Med Teach*. 2011;33(11):875-886. https://doi.org/10.3109/0142159X.2011.595434
