# Supplementary material for: Clinical Skills Tutoring Program (CSTP): Developing a Curriculum for Medical Student Clinical Skills Peer Tutors
Source: MedEdPORTAL. 2022 Feb 14;18:11225. doi: 10.15766/mep_2374-8265.11225 (PMC8841391; doi:10.15766/mep_2374-8265.11225)
Supplement: Supplementary file 1 — Tutor Curriculum Learning Objectives and Content.docxTutor Curriculum Supplement.docxTutor Curriculum Nuts and Bolts.docxTutor Checklist.docxCSTP Facilitator Guide for Tutor Training Session.docxTutor Training Session Survey.docxTutor Participant Survey.docxStudent Participant Survey.docx [file mep_2374-8265.11225-s001.zip › C. Tutor Curriculum Nuts and Bolts.docx]

**Clinical Skills Tutoring Program (CSTP)**

**Nuts and Bolts: Logistics and How-To Tips**

*Welcome to the Clinical Skills Tutoring Program! This document will provide some details to help you get started with organizing meetings and beginning your work as a tutor. In addition, this document will help clarify some details on logistics and expectations not covered elsewhere.*

**Part 1. Sequence of Events**

As a tutor for this program, you will move through a sequence of events. See checklist document for details.

**Reaching out to the Student**

As a tutor, you will be notified once you are matched with a student through email. Please reach out to your student within 2 days of receiving the email. You should send an email to your student introducing yourself and setting up a first meeting date. An example of this email is the following:

*“Hi (insert Student name),
I am (insert your name), a fourth-year medical student who has been assigned as your peer tutor in the Clinical Skills Tutoring Program. I would like to schedule a first meeting with you to get to know you better and also to plan our coaching sessions together. Please let me know what date and time would work best for you for a 1 hour timeframe.* *Here are some times I have available in the next two weeks: ***. We will be meeting virtually over Zoom at this time. I can set up a calendar invite with a zoom link for us. I am looking forward to meeting you!*

*Sincerely,*

*(insert your name)”*

During your email exchange with the student, always remain professional.

**Discuss the student with the student’s coach**

Please arrange a meeting with the student’s coach prior to the start of the tutoring sessions. Because the CMC coaches are core teachers and observers of clinical skills as well as the primary source of feedback for students in their pre-clinical training, this meeting is essential to help organize areas of improvement for the students and how the student receives feedback. There will be situations where reaching out to the student’s coach with the student involved may be best in terms of building trust with your student. We recommend discussing the best approach with one of the CSTP co-directors. An example of the email to the student coach is the following:

*“Hi (insert Student’s coach’s name),*

*I am (insert your name), a fourth-year medical student who will be working with (insert student’s name) as part of the Clinical Skills Peer Tutoring Program. Before meeting with (insert student’s name) it would be great if I could talk with you to discuss specific areas of improvement, the student’s learning style, and best methods on providing feedback. Please let me know what date and time would work best for you to chat over the phone for 15-20 minutes. Here are some times I have available in the next two weeks: ***. Thank you very much for your time*

*Sincerely,*

*(your name).”*

**Meet with the student for tutoring sessions**

Please meet with your student for at least one session every month. The number of sessions will depend on the needs identified by the student and/or suggested by the tutor or program directors. The sessions will be one-on-one and can consist of sessions with standardized patients. There will set times when standardized patients are available for tutor-student pairs to work with.

Key goals for the one-on-one sessions include, but are not limited to:

1. Check-in with your student on a personal level to get to know your student better and to explore what barriers/challenges the student may be facing beyond your work with them.
2. Creating and checking-in on a schedule for tutoring sessions, continuing or adjusting the expected timeline as needed
3. Analyze and discuss prior performance reports and recorded videos related to clinical skills
4. Formulate an ILP with the student, design a customized schedule, and implement a coaching plan with the student to achieve the goals of the ILP
5. Deliver effective feedback on self-efficacy, goal setting, strategic planning, self-monitoring, self-evaluation, attribution beliefs, and adaptive changes based on direct observations of clinical skills performance
6. Model proficiency in hypothesis-driven history taking and physical exam skills, diagnostics/clinical reasoning, and communication skills relevant to commonly encountered symptoms and diagnoses

Key goals for the standardized patient sessions may include:

1. Observation and feedback on history-taking
2. Observation and feedback on physical exam
3. Observation and feedback on interpersonal and communication skills
4. Getting comfortable with the room layout, equipment, and test day environment
5. Practicing full encounters with a standardized patient

**Requesting a Virtual Standardized Patient**

During the COVID-19 pandemic, the opportunity to utilize standardized patients in the Kanbar Center is not going to be possible for now. We have incorporated the opportunity for groups to request the use of virtual standardized patients to supplement their tutoring sessions.

If you and your student are interested in practicing with a virtual standardized patient, please contact the program directors. In addition, we will check-in with all tutor groups once every month and schedule virtual standardized patient days if groups are interested. When scheduling a virtual standardized patient, please provide the program directors with the following information:

1. The available days and times in the next 3-4 weeks for you and your student
2. The level of training of your student and any courses they have not taken (e.g. BMB, Life Stages)
3. What standardized patient cases has your student completed in the past?
4. What specific clinical skill(s) is your student currently practicing?

You and your student will be notified of the time and date for the standardized patient session and will be sent the information for the Zoom meeting. In addition, you will be provided with the case information and case checklist ahead of time to prepare for the session. These are for your own preparation and should not be shared to your student prior to the session. In addition, please do not share these among your peers as these cases may be re-used for clinical examinations in the future.

**Working with a Virtual Standardized Patient**

The virtual standardized patient will mimic a normal patient encounter. We will outline what to expect in the virtual encounter and the workflow so that you can help keep help your student stay on track during the encounter.

1. The Zoom meeting will be scheduled for a duration of 1-1.5 hours for two patient cases
2. You will join the call with your student with one or two standardized patients in the meeting.
3. We recommend spending 5 minutes to check-in with your student and formulate a plan, including which case to do first and whether to do feedback after each individual case or after all the cases are completed.
4. You will work with one standardized patient at a time. The standardized patient for the second case is aware of the waiting time and will mute their video and microphone until given directions to unmute.
5. When the case starts, please read the “Door Notes” located in the case documents out loud to your student.
6. To do a physical exam, the student may tell the standardized patient, for example, “I will now listen to your lungs in six places”, where the patient will then respond with “crackles in the right lower base” or “clear to auscultation”. You may provide this guidance to your student before the clinical encounter begins.
7. Please allow time for feedback provided by you and the standardized patient. You may decide whether to use the scoring system provided by the checklists to track student performance.
8. If needed, we recommend groups to spend more time on giving feedback and continuing discussion after the allotted time with the SPs. This can be done in your separate Zoom meeting.

**Completing a check-out with program directors**

At the end of your work with the student, please contact the program directors to review tutoring experience. Please note that you can check in with program directors after any session if you have questions or concerns. You will be assigned a primary program director as a point of contact throughout your involvement in the program.

During this phone meeting, the following topics will be discussed:

- Any benefits or learning accomplishments gained during tutor experience
- Any perceived challenges or negative experiences during tutor experience
- Reflection of experience (has experience motivated you to teach in the future?)
- Feedback on the experience (what went well, what didn’t go so well, how can we improve?)
- Discuss the student’s performance and growth
- Discuss the tutor’s performance and growth
- Completion of tutor participation survey

**Completing a check-out with student’s coach**

At the end of the sessions with the student, please contact the student’s coach via email to set up a meeting in-person or over the phone to review tutoring experience. As noted above for initial meeting, there may be situations where doing this check-out along w/ your student (or including them in email communication) will be best. During this meeting, the following topics will be discussed:

1. Student ILP and Learning Goals – their achievements and future goals
2. Specific areas categorized as strengths
3. Specific areas categorized as areas needing improving – discuss progress and milestones
4. Student’s current clinical skills performance compared to prior
5. Student’s future learning goals
6. Discussing how the coach can help implement future learning goals and reinforce student’s progress

A summary of tutoring progress consists of keeping track of the following elements:

1. Student Meetings

- How many meetings have been scheduled
- Length of each meeting
- Topics discussed in each meeting
- Teaching materials based on student’s preferred learning method

1. Student Progress

- Performance on standardized patient cases
- Specific areas that have shown improvement

1. ILP and SMART goals

- Progress on SMART goals
- Evolution of ILP over time
- Future Learning Goals and Next Steps

Because there will be a lot of information shared and received during each meeting and with more SMART goals and ILP forms submitted over time, stay organized and use the above when summarizing tutoring progress to a program director or to the student’s coach. The notes listed above will help in providing the material and examples during the discussion.

*If you find yourself having difficulty with any of these tasks, please reach out to the program directors who can provide further assistance.*
