# Supplementary material for: Clinical Skills Tutoring Program (CSTP): Developing a Curriculum for Medical Student Clinical Skills Peer Tutors
Source: MedEdPORTAL. 2022 Feb 14;18:11225. doi: 10.15766/mep_2374-8265.11225 (PMC8841391; doi:10.15766/mep_2374-8265.11225)
Supplement: Supplementary file 1 — Tutor Curriculum Learning Objectives and Content.docxTutor Curriculum Supplement.docxTutor Curriculum Nuts and Bolts.docxTutor Checklist.docxCSTP Facilitator Guide for Tutor Training Session.docxTutor Training Session Survey.docxTutor Participant Survey.docxStudent Participant Survey.docx [file mep_2374-8265.11225-s001.zip › D. Tutor Checklist.docx]

**Clinical Skills Tutoring Program (CSTP)**

**Tutor Workflow and Checklist**

*As a tutor for this program, you will move through the following sequence of events. This checklist will keep you organized on which tasks you have completed.*

- - Get notice of an assigned student you will work with
  - Connect with one of the Program Directors of the Clinical Skills Peer Tutoring Program to discuss the assigned student
  - For some students: Review the student’s prior reports and videos related to their clinical skills
  - Reach out to the student’s coach
    1. Introduce yourself
  - Set up a time to discuss the student with the student’s coach (note: program directors will help guide you on best way to have coach conversation, e.g., with your student, with program director etc.)
  - Reach out to the student
    1. Introduce yourself
    2. Set up a time/place for the first meeting
  - Review relevant sections of CLE and tutor curriculum for preparation
  - Meet with the student for tutoring sessions
    1. One on one coaching sessions
    2. Working with standardized patients
    3. Other activities targeted to the student’s learning goals
  - Review and discuss the student’s Individualized Learning Plan (ILP) before and after each session.
  - Submit a single, most-recent ILP for your student to the program co-directors each month or at least every 4 sessions.
  - Complete all tutoring sessions with the student, check in with program co-directors as needed between tutoring sessions or during monthly tutor support sessions
  - Track your hours and submit hours monthly via Qualtrics survey to ensure prompt payment for your completed hours
  - Complete a check-out with the tutoring program co-directors
  - Complete a check-out with the student’s coach to provide a summary of the tutoring accomplished
  - Complete a Tutor Participation Survey
  - Repeat steps for each assigned student

*If you find yourself having difficulty with any of these tasks, please reach out to the program directors who can provide further assistance.*
