# Supplementary material for: Clinical Skills Tutoring Program (CSTP): Developing a Curriculum for Medical Student Clinical Skills Peer Tutors
Source: MedEdPORTAL. 2022 Feb 14;18:11225. doi: 10.15766/mep_2374-8265.11225 (PMC8841391; doi:10.15766/mep_2374-8265.11225)
Supplement: Supplementary file 1 — Tutor Curriculum Learning Objectives and Content.docxTutor Curriculum Supplement.docxTutor Curriculum Nuts and Bolts.docxTutor Checklist.docxCSTP Facilitator Guide for Tutor Training Session.docxTutor Training Session Survey.docxTutor Participant Survey.docxStudent Participant Survey.docx [file mep_2374-8265.11225-s001.zip › E. CSTP Facilitator Guide for Tutor Training Session.docx]

**UCSF School of Medicine**

**Clinical Skills Tutoring Program (CSTP)**

**Orientation and Training**

Facilitator’s Guide

**Participants:** School of Medicine MS4s selected as CSTP tutors

**Faculty**: CSTP Co-directors (2 faculty members)

**Format**: 2-hour, small group, in-person or virtual session

**Session Outline:**

**Introductions/Check in** (15 minutes)

1. Share something you are proud of from the past month, personally or professionally (modeling a check in that you could do w/ your student)
2. What is one thing you would like to learn as a tutor during your participation in the CSTP?

**Welcome from CSTP Co-Directors: focus on big picture** (not a review of learning objectives at this point in learning lessions) (10 minutes)

**Key Points to share:**

- You can make a big impact, be a truly trusted mentor to a peer or near peer student. Your applications highlighted your own strong clinical skills development and your commitment to teaching/mentoring – we were extremely impressed.

- This program is meeting an important need in the School of Medicine, to expand capacity for clinical skills practice for students who need more time/resources to achieve competence in clinical skills.

-Examples of students you are tutoring can be 1^st^ to 4^th^ year medical students who are referred by coaches, deans, clerkship directors, or faculty who manage clinical skills exams, clerkship remediation, and sub-internship remediation.

-Confidentialty, keeping and maintaining trust of the student is critical, getting extra help is a vulnerable thing to do. You have to hold this responsibility.

- You are not alone as tutors, you always have us to reach out to, we always want you to reach out if you have any questions or anything comes up that you are not sure how to handle.

-We will have tutor support and training sessions occurring monthly facilitated by us and other faculty for skill building, community building, and for asking questions.

- We value and appreciate your feedback at every step – we want to iterative improve our program.

-Expectations for time commitment (emphasize that this varies based on specific pairings). In the past, some pairings have met every week while others met once a month.

**Review of CSTP Nuts and Bolts document, Q&A about logistics** (15 minutes)

**-**Open and review documents together

-Discuss the need to submit Individualized Learning Plans (ILPs). We encourage ILP submission once monthly or at least every 4th session. Send via email to CSTP program directors.

**Review of tutor curriculum** (20 minutes)

**Review big picture of curricular components:**

- Orientation/training session today with microskills practice

- Tutor Curriculum document w/ specific objectives

- Introduce tutor curriculum document, have tutors pull up current version, let tutors know most updated version will always be on CLE and we will send them link to CLE after today’s session

- Review objectives, let tutors know that objectives follow the arc of the experience, core framework on which the curriculum is built

- Review structure of tutor curriculum document - key points for each objective are there, key articles to read and reflection questions for self-assessment, appendix for further learning on objectives

-Deep dive into self-regulated learning, using the tutor curriculum document as a guide. Model specific phrases or strategies used in feedback of this model and encourage practice during break-out sessions. (Tables 1 and 2 in Objective IV).

- CLE page w/ tutor curriculum plus additional resources for each clinical skills domain

- If students have a great resource- share it with us and we can add it to our collection.

**BREAK** (5 minutes)

**Microskills practice** (45 minutes total)

**Microskill 1** (25 minutes): Practice having conversation with a student re: prior performance, learning goals, and the Individualized Learning Plan (ILP)

- Introduce framework in tutor curriculum – strengths, areas for growth, appreciative inquiry framework
- Share an ILP sample document (see Appendix 1 below) for break-out activity.
- Break out: in Break out rooms (virtual session) or in different corners of the same room (in-person session) ask tutors to practice meeting their student for the first time, discussing learning goals and reviewing ILP sample with student, make goals “SMART” or “SMARTer”, and form a plan to achieve goals. (7.5 minutes for each person = 15 minutes total) – we will announce when pairs should switch!
- Coming back together: Discuss what went well, what skills you used, what you found difficult? (5 minutes)

**Microskill 2 (20 minutes):** Practice providing feedback on sandardized patient encounter or role play of encounter

- Review tutor curriculum materials briefly re: feedback
- CSPT co-directors role play a brief patient encounter (one faculty member pretends to be a patient w/ a cough x 3 months. The other faculty member is the student. Share that the overall learning goals for the student are to (1) To always start the encounter with open ended questions and (2) To follow a systematic approach to HPI such as OLDCARTS every time.
- Further details on the role play for facilitators:
  - The patient is Ms. Taylor, a 65 yo woman with 50 pyh of smoking, h/o COPD, presenting with 3 months of productive cough w/ occasional blood tinged sputum and 20 lbs weight loss over 3 months.
  - Provider starts questions with “How much do you smoke”, “How long have you smoked?” “When do you have the cough?” “What have you tried for the cough?” “Is there anything else I should know about the cough?” Model a student bouncing around from family history to PMH to meds, etc (ie a disorganized HPI).
  - Run role play only for about 3 minutes
- After the encounter, ask tutors to taking turns being in the “hot seat” and role play actually giving feedback/debriefing with the student about the encounter (e.g., round robin style).
- Facilitator acknowledges how awkward and challenging this may feel

**Wrap-up and check out (10 minute):** thank tutors for their time, ask each tutor to share one key learning point from the session by either sharing out loud or adding to the chat box (if a virtual session)

**Appendix 1: Sample ILP document**

**Clinical Skills Learning Goal 1:**

I want to get better at the physical exam.

**Feedback/data I have received related to this goal (e.g., what supports the decision to choose this goal):**

I got a very low score on my DOCS2 exam for physical exam.

**Plan for how I will work on Learning Goal 1 with my tutor:**

I want to practice the physical exam with standardized patients.

**Clinical Skills Learning Goal 2:**

I want to build better rapport with patients in the next month.

**Feedback/data I have received related to this goal (e.g., what supports the decision to choose this goal):**

I got comments from the SPs during DOCS2 that I wasn’t connecting well. I have also been told by a friend that I do not make good eye contact when listening to her.

**Plan for how I will work on Learning Goal 2 with my tutor:**

I hope to make better eye contact with SPs and patients when they are speaking so that I can connect better with patients. I will focus less on taking long notes and more on listening. I want my tutor to give me feedback on how I do this during the next month.

**Clinical Skills Learning Goal 3:**

I want to get better at history-taking.

**Feedback/data I have received related to this goal (e.g., what supports the decision to choose this goal):**

I received comments that I spent too much time on history-taking on my DOCS2 exam and did not come up with a good list of possible diagnoses.

**Plan for how I will work on Learning Goal 3 with my tutor:**

I hope to learn how and what questions to ask for common chief complaints, but I don’t have a specific plan yet.
