# Supplementary material for: Clinical Skills Tutoring Program (CSTP): Developing a Curriculum for Medical Student Clinical Skills Peer Tutors
Source: MedEdPORTAL. 2022 Feb 14;18:11225. doi: 10.15766/mep_2374-8265.11225 (PMC8841391; doi:10.15766/mep_2374-8265.11225)
Supplement: Supplementary file 1 — Tutor Curriculum Learning Objectives and Content.docxTutor Curriculum Supplement.docxTutor Curriculum Nuts and Bolts.docxTutor Checklist.docxCSTP Facilitator Guide for Tutor Training Session.docxTutor Training Session Survey.docxTutor Participant Survey.docxStudent Participant Survey.docx [file mep_2374-8265.11225-s001.zip › Educational_Summary_Report_11225.pdf]

MedEdPORTAL® | The AAMC Journal of Teaching and Learning Resources

Original Publication

Open Access

# Clinical Skills Tutoring Program (CSTP): Developing a Curriculum for Medical Student Clinical Skills Peer Tutors

Chih-Chiun Jamie Chang, MD*, Sara-Megumi Rumrill, MD, Abigail Phillips, MD

*Corresponding author: jamiechang@live.com

## Abstract

**Introduction:** There are few curriculum materials designed to provide training and support for peer tutors to become effective clinical skills teachers. We designed the Clinical Skills Tutoring Program (CSTP) curriculum to guide tutors to help their students reflect on clinical skills performance, create an individualized learning plan, and engage in improvement based on feedback to achieve clinical skills competencies. **Methods:** Curriculum content was delivered through an in-person training session, formal curriculum written content, online resources, and longitudinal support from faculty directors. Tutors (fourth-year medical students) received surveys to evaluate the in-person training session, curriculum resources, and overall program experience. Student participants (medical students of any year) completed a survey to rate their satisfaction in working with their tutors. **Results:** There were 12 tutors in cohort 1 and 18 tutors in cohort 2. Survey response rates ranged from 50% to 70% among tutors. The tutors were satisfied with the in-person training session, program experience, curriculum resources, support from directors, development of learning goals with the student, and clinical skills practice with the student (mean Likert ratings greater than 4 out of 5). Student participants were satisfied with their experience creating learning goals and receiving feedback from their tutors. **Discussion:** The tutor curriculum fills a gap by training and supporting tutors before and during their work with students needing further resources and remediation in one or more clinical skills domains. The curriculum can be implemented and further adapted by other tutoring programs locally and nationally.

### Keywords
Tutoring, Curriculum Development, Peer Teaching, Remediation, Self-Regulated Learning, Feedback

## Educational Objectives

By the end of the tutor curriculum, tutors will be able to:

1. Define learning theories and processes that support peer tutoring, including cognitive congruence, social congruence, self-regulated learning, and the master adaptive learner conceptual model.
2. Analyze and discuss prior performance reports and recorded videos related to clinical skills with a student.
3. Describe the components of an individualized competency-based learning plan (ILP), formulate an ILP with the student, design a customized schedule, and implement a coaching plan with the student to achieve the goals of the ILP.
4. Deliver effective feedback on self-efficacy, goal setting, strategic planning, self-monitoring, self-evaluation, attribution beliefs, and adaptive changes to a student based on direct observations of clinical skills performance.
5. Model proficiency in hypothesis-driven history-taking and physical exam skills, diagnostics/clinical reasoning, and communication skills relevant to commonly encountered symptoms and diagnoses.
6. Display effective skills in communication and coordination with program directors and student coaches to provide coordinated support to the student.

**Citation:**
Chang CCJ, Rumrill SM, Phillips A. Clinical Skills Tutoring Program (CSTP): developing a curriculum for medical student clinical skills peer tutors. *MedEdPORTAL*. 2022;18:11225.
https://doi.org/10.15766/mep_2374-8265.11225

## Introduction

In medical school, students are longitudinally assessed on clinical skills performance with standardized patient assessments[1] and clinical rotations. Some students require additional time and practice to achieve clinical skills competencies and may benefit from remediation. Remediation is a complex and time-intensive process that involves diagnosing the learner while exploring potential social and emotional concerns.[2,3] Incorporating a peer

Copyright © 2022 Chang et al. This is an open-access publication distributed under the terms of the Creative Commons Attribution license.

teaching program can supplement existing faculty resources and increase the bandwidth for one-on-one instruction in remediation. By featuring a novel formalized curriculum designed to facilitate peer tutoring through goal setting and support, this program addresses an important gap in the literature: the role of peer coaching on clinical skills for students in need of further skills development.

Peer tutors can play a valuable role in assisting medical student remediation by utilizing shared cognitive and social experiences to create a safe, inclusive learning environment where the student feels more confident to ask questions, share concerns, and make mistakes. Studies find that students feel more comfortable and have their personal struggles better understood when working with a peer tutor,[4] resulting in increased confidence,[5] engagement, and ownership of their learning.[6] Peer tutor programs have been found to be beneficial in the development of medical knowledge,[7] clinical skills,[5] and student self-efficacy.[8] These programs also benefit peer tutors by improving their skills in teaching and communication while solidifying their own clinical knowledge.[7,9] At our institution, the primary drivers for the development of this program were (1) literature as described above supporting peer tutoring, (2) interest in studying how the benefits of peer tutoring could be applied in a novel context of clinical skills peer tutoring for remediation and skill building, and (3) a need to augment clinical skills resources beyond faculty availability. Faculty coaches have roles that include other tasks, such as career mentorship and facilitating systems improvement projects, so it is important to be able to offer additional clinical skills resources to a subset of students who require added time and coaching to achieve competence in one or more clinical skills domains.

Peer tutor programs do not consistently provide training to their tutors.[10,11] This makes it challenging for tutors to obtain skills in self-reflection and to understand how to become an effective clinical teacher.[12] Peer tutors may also receive inadequate instruction on core teaching skills essential to remediation such as delivering effective feedback.[13] Prior published versions of peer tutor curricula incorporate teaching and feedback models adapted for problem-based learning[14] and preclinical content.[15,16] When it comes to clinical skills, which we define as skills in conducting patient encounters including history taking, physical exam, professional communication, and diagnostic reasoning, there are few formal curriculum materials designed for peer teaching. We proposed a curriculum that would guide medical student peer tutors in helping their students achieve successful remediation by reflecting on past clinical skills performance, creating an individualized learning plan, and adapting their strategy based on feedback, specifically in the realm of clinical skills.

We conducted a focused literature review of teaching and learning theories supporting the use of peer tutors and found to be beneficial in student remediation. This served as the theoretical backbone of our curriculum. A needs assessment was conducted using semistructured focus groups with key stakeholders, including medical education faculty experts, departmental leadership, and clinical coaches, to develop the six core educational objectives of the curriculum. At our institution, medical students are paired with longitudinal clinical faculty coaches who serve as direct teachers and mentors for all 4 years. These coaches are the initial instructors for clinical skills and play an important role in observing and providing early feedback to students. The focus group interviews were conducted not only to elicit perspectives from faculty with expertise in this area but also to ensure that tutors would be trained in teaching skills and serve as tutors in a way that was aligned and collaborative with structures and resources already existing in the medical school for the student.

We designed the Clinical Skills Tutoring Program (CSTP) to provide curriculum materials, formal training, and longitudinal support for peer tutors to be effective clinical skills teachers to medical students referred to the program for remediation. We hypothesized that the peer tutors would be satisfied with the curriculum and that the students would be satisfied with their experience working with formally trained tutors.

## Methods

### Participants

Fourth-year medical students were invited to apply to be tutors. The application survey focused on assessing their clinical skills, personal teaching experiences, and faculty references to gauge prior clinical performance and teaching abilities. In this publication, tutors are defined as the applicants who were subsequently selected by faculty leadership based on review of their application and who participated in the program. Student participants were medical students from all years of training referred to our program by faculty leadership and attending physicians. Top reasons for referral included a nonpassing grade for a clinical rotation (preceptorship, core clerkship, subinternship), an unsatisfactory performance on standardized clinical examinations, difficulties in acquiring clinical skills milestones as observed by faculty, or extended length of time away from clinical medicine (e.g., leave of absence). Unlike the

Copyright © 2022 Chang et al. This is an open-access publication distributed under the terms of the Creative Commons Attribution license. 

first cohort, which included only referred students, the second cohort included students who were invited to self-refer to the program if they could identify specific learning goals in clinical skills domains. This was a change we implemented due to the COVID-19 pandemic and reported needs among students who had fewer clinical skills learning opportunities.

Tutors were either true peers (same year in medical school) or, more commonly, near-peers (1-3 years apart in medical school) to the student participants. Tutors were compensated $20 per hour for training sessions, preparation time, and tutoring time. Paid preparation time was limited to 1 hour for every 2 hours of tutoring time. Tutors were expected to meet with their student approximately twice a month for 2-4 months (the frequency and duration varied depending on the learning goals of the student), and the amount of contact with the student's coach was variable depending on the needs/wishes of the student. Tutors made a 1-year commitment when they applied to the program, and they shared their availability for the year (e.g., tutors were not expected to be available during busy clinical rotations). At the end of the second cohort, one tutor was selected to be the Lead Tutor and was paid for an additional 5-10 hours per month to provide administrative support to the program. The program was not part of an elective; it was a paid extracurricular activity.

### Curriculum Content

The formal tutor curriculum written content (Appendix A) contained learning materials, references, and examples for tutors to be successful in achieving the core educational objectives for this program. The curriculum introduced tutors to concepts such as social and cognitive congruence,[17] two main learning theories that supported peer tutoring by teaching how to establish rapport and trust with students. Tutors also received training on learning theories supporting student remediation, including the self-regulated learner[18] and master adaptive learner models.[19] Additionally, tutors were instructed on the importance of confidentiality in this program. For example, names of students were never discussed during monthly tutor learning/community-building sessions, and tutors learned that both the tutor and the student were emailed separately to confirm that the pairing was working (e.g., no dual relationship/conflict of interest) prior to finalizing each pairing. The formal curriculum was supplemented with additional documents. A tutor curriculum supplement (Appendix B) provided further figures and examples to augment the core reading material. The nuts and bolts document (Appendix C) was created to offer helpful tips for new tutors in navigating the logistics of the program, such as reaching out to the student or communicating with program directors. Lastly, a tutor checklist (Appendix D) was designed to consolidate the anticipated workflow of a peer tutor and the tasks they were expected to complete in the arc of the relationship with the student.

We identified increased peer collaboration and information sharing as an area for improvement based on our results collected from the first cohort of tutors. For our second cohort, we incorporated formal monthly tutor community-building sessions to increase the opportunities for teaching skills development, information sharing, and peer support. These sessions were facilitated by program directors and discussed topics decided on among tutors as high yield in working with their students, such as how to provide high-quality feedback.

### Delivery of Curriculum Content

The tutor curriculum materials were delivered to the tutors through multiple modalities: an initial in-person tutor training session, formal tutor curriculum written content, online resources (including clinical skills educational materials), and longitudinal support from faculty directors. The in-person tutor training session was conducted prior to tutors being matched with a student and was mandatory. This 2-hour session provided an overview of program goals, expected tutor responsibilities, tutor curriculum documents, and how to access learning materials, as well as micro-skills practice on content related to core learning objectives including reviewing performance reports, refining an individualized learning plan, and providing feedback to a student (Appendix E: facilitator guide). During the session, the tutors received the formal tutor curriculum written content and received access to the Collaborative Learning Environment (CLE), an online webpage containing files, documents, and links providing instruction on each of the educational objectives. The page was designed as an easy-to-access online space where tutors could revisit tutor curriculum materials and access supplemental reading materials taking a deeper dive into the educational objectives. In addition, the CLE housed teaching materials for clinical skills (e.g., history taking, physical exam, and diagnostic reasoning) for tutors to share with their students, while providing a platform for tutors to share teaching materials they developed or found to be beneficial. The CLE had links to common resources such as Bates physical exam videos, Step 2 CS materials (prior to the elimination of this exam), and school-specific clinical skills learning content. Each student-tutor pair was assigned a private online folder where students shared their learning plans, tutors provided additional teaching materials, and any other content could be stored or exchanged between

Copyright © 2022 Chang et al. This is an open-access publication distributed under the terms of the Creative Commons Attribution license.

the pair. Lastly, tutors received longitudinal support from faculty directors through email and phone check-in meetings.

Evaluation Methodology

Evaluations were completed for cohort 1 (January 2020-May 2020) and cohort 2 (June 2020-December 2020). Two surveys were distributed to the tutors to evaluate the tutor curriculum and training materials. The first survey evaluated the usefulness of components of the initial in-person training session and was given to tutors within 1 week following the session (Appendix F). Tutors received a second survey after completion of their participation in the program (May 2020 for cohort 1, December 2020 for cohort 2) to evaluate their satisfaction with specific components of the curriculum, program support, and development of teaching skills with the student (Appendix G); this was sent within days to weeks of completing work with their student. After concluding their work with a tutor, the student participants received a survey (Appendix H) that evaluated their satisfaction with the overall program experience, relationship with their tutor, development of learning goals with the tutor, support from program directors, effectiveness of their tutor in providing clinical skills coaching, and clinical skills practice with the tutor. All surveys used 5-point Likert scales and contained additional space for free-response feedback. All survey links and responses were anonymous.

Additional input for the purposes of program improvement was obtained from tutors through a semistructured focus group. The aims of the focus group were to (1) explore the tutor's personal experience of working as a peer tutor; (2) elicit feelings related to training, mentorship, and resources available for tutors; and (3) gather feedback on program strengths and areas for improvement. We reviewed the transcript of the focus group discussion for the purposes of programmatic evaluation; the goal was to identify strengths and areas for continuous quality improvement in the program and tutor curriculum. This project was deemed exempt by the University of California, San Francisco, Institutional Review Board.

## Results

The first cohort of the CSTP began in January 2020. We received a total of 15 tutor applications and selected 10 fourth-year medical students as peer tutor participants. For the second cohort starting in June 2020, we received 25 tutor applications and expanded the program to select 18 tutors. Tutors were selected based on survey responses that demonstrated interest in medical education and teaching, prior experiences as a teacher, and excellence in clinical skills. In the first cohort, 12 medical students from years 1-4 were referred by faculty to the program as student participants for clinical skills remediation. Tutors spent an average of 7-10 hours per month dedicated to the program when they were paired with a student, with an average of 4-6 hours per month spent on direct tutoring and 3-4 hours per month spent on training and preparation.

In the first cohort, five of 10 tutors responded to the in-person training survey. In the second cohort, 12 of 18 tutors responded to the survey. Tutors from both the first and second cohorts found the components of the training session very to extremely useful. Overall, the training session received an average rating of 4.5 (1 = *not at all useful,* 5 = *extremely useful*; 4.5 is between very and extremely useful). Facilitation of the session (average rating: 4.8, between very and extremely useful), training session materials (average rating: 4.6, between very and extremely useful), discussion of tutor curriculum and objectives (average rating: 4.6, between very and extremely useful), and micro-skills practice sessions (feedback simulation average rating: 4.6, between very and extremely useful; reviewing learning plan average rating: 4.6, between very and extremely useful; and reviewing score report average rating: 4.6, between very and extremely useful) all received evaluations that suggested the tutors found the components useful for preparation (Table 1).

Following the tutors' involvement in the program, we received survey feedback on the program experience and tutor curriculum from seven of 10 tutors in the first cohort and 12 of 18 tutors in the second. The average rating of the overall experience as a tutor was 4.7 (1 = *extremely dissatisfied,* 5 = *extremely satisfied*). The majority of tutor curriculum components received ratings of 4.0 and above, suggesting broad satisfaction (Table 2). The lowest rating of 3.6 was identified in the first cohort when evaluating opportunities for community building with other tutors, suggesting this as a key area for improvement. After implementing community-building sessions for our second cohort of tutors, the average rating improved to 4.4, suggesting greater tutor satisfaction.

We received survey responses and feedback from five of 12 student participants in the first cohort and 11 of 33 in the second cohort. The average rating of the overall program experience was 4.9 (1 = *extremely dissatisfied*, 5 = *extremely satisfied*). Student participants were satisfied with their relationship with tutors (average rating: 4.8). In addition, students were satisfied with how trained tutors approached clinical skills remediation through development of learning goals (average rating: 4.9), delivering clinical skills coaching (average rating: 4.9), and facilitating clinical skills practice (average rating: 4.5; Table 3).

Copyright © 2022 Chang et al. This is an open-access publication distributed under the terms of the Creative Commons Attribution license.

**Table 1.** Peer Tutor Ratings of In-Person Training Session (Cohort 1 $n$ = 5, Cohort 2 $n$ = 12)

| | No. of Respondents | | | | | |
|---|---|---|---|---|---|---|
| Session Component[a] | Extremely Useful | Very Useful | Moderately Useful | Slightly Useful | Not at All Useful | Average Rating |
| Overall in-person training session | | | | | | |
| Cohort 1 | 4 | 1 | 0 | 0 | 0 | 4.8 |
| Cohort 2 | 5 | 6 | 1 | 0 | 0 | 4.3 |
| Cohorts 1 + 2 | 9 | 7 | 1 | 0 | 0 | 4.5 |
| Overview of the program | | | | | | |
| Cohort 1 | 5 | 0 | 0 | 0 | 0 | 5.0 |
| Cohort 2 | 6 | 5 | 0 | 1 | 0 | 4.3 |
| Cohorts 1 + 2 | 11 | 5 | 0 | 1 | 0 | 4.5 |
| Curriculum objectives and discussion of tutor curriculum | | | | | | |
| Cohort 1 | 4 | 1 | 0 | 0 | 0 | 4.8 |
| Cohort 2 | 8 | 2 | 2 | 0 | 0 | 4.5 |
| Cohorts 1 + 2 | 12 | 3 | 2 | 0 | 0 | 4.6 |
| Micro-skills practice—reviewing score report[b] | | | | | | |
| Cohort 1 | 3 | 2 | 0 | 0 | 0 | 4.6 |
| Micro-skills practice—reviewing individualized learning plan | | | | | | |
| Cohort 1 | 3 | 2 | 0 | 0 | 0 | 4.6 |
| Cohort 2 | 9 | 1 | 2 | 0 | 0 | 4.6 |
| Cohorts 1 + 2 | 12 | 3 | 2 | 0 | 0 | 4.6 |
| Micro-skills practice—interview simulation and feedback | | | | | | |
| Cohort 1 | 3 | 1 | 1 | 0 | 0 | 4.4 |
| Cohort 2 | 9 | 2 | 1 | 0 | 0 | 4.7 |
| Cohorts 1 + 2 | 12 | 3 | 2 | 0 | 0 | 4.6 |
| Organization of session | | | | | | |
| Cohort 1 | 4 | 1 | 0 | 0 | 0 | 4.8 |
| Cohort 2 | 7 | 4 | 1 | 0 | 0 | 4.5 |
| Cohorts 1 + 2 | 11 | 5 | 1 | 0 | 0 | 4.6 |
| Training session materials | | | | | | |
| Cohort 1 | 4 | 1 | 0 | 0 | 0 | 4.8 |
| Cohort 2 | 8 | 3 | 1 | 0 | 0 | 4.6 |
| Cohorts 1 + 2 | 12 | 4 | 1 | 0 | 0 | 4.6 |
| Facilitation of session | | | | | | |
| Cohort 1 | 5 | 0 | 0 | 0 | 0 | 5.0 |
| Cohort 2 | 10 | 1 | 1 | 0 | 0 | 4.8 |
| Cohorts 1 + 2 | 15 | 1 | 1 | 0 | 0 | 4.8 |

[a]Rated on a 5-point Likert scale (1 = *not at all useful,* 5 = *extremely useful*).
[b]Only conducted in cohort 1.

Feedback collected from focus groups (six participants in the first focus group, two participants in the second) identified key themes of strengths and areas for improvement in the tutor curriculum and program design that helped in guiding the evaluation and future direction of the program (Table 4). The tutors noted curriculum structure, director support, and learning resources as three content areas that contributed positively to their development as teachers and to their ability to achieve the core educational objectives. Tutor suggestions for improvements in the curriculum design included incorporating opportunities for tutor check-ins, community building, collaboration, and information sharing. Tutors felt that learning from their colleagues' experiences and sharing strategies in working with students could further improve their personal growth as educators. Tutors also requested access to more specific resources to share with their students, especially in preparation for inpatient third-year rotations.

## Discussion

The CSTP introduced a tutor curriculum providing formal training and longitudinal support for peer tutors to become effective clinical skills teachers for other medical students. The tutors were trained to achieve six core educational objectives that involved developing effective skills in goal setting and providing iterative feedback to facilitate their students' growth into self-regulated learners. Under the guidance of their tutors, students learned strategies to create and update their individualized learning plan.[20] In our program, tutors were satisfied with the in-person training session and tutor curriculum documents, while student participants were satisfied with their experience working with trained tutors.

The response rate for surveys was lower than anticipated. The lower response rate was due to multiple factors; timing of surveys was likely a significant driver of this. For example, surveys

Copyright © 2022 Chang et al. This is an open-access publication distributed under the terms of the Creative Commons Attribution license.

**Table 2.** Peer Tutor End-of-Program Survey Ratings of Tutor Curriculum and Program Components (Cohort 1 $n = 7$, Cohort 2 $n = 12$)

| Question[a] | No. of Respondents | | | | | Average Rating |
|---|---|---|---|---|---|---|
| | Extremely Satisfied | Somewhat Satisfied | Neutral | Somewhat Dissatisfied | Extremely Dissatisfied | |
| Overall experience as a peer tutor | | | | | | |
| Cohort 1 | 5 | 2 | 0 | 0 | 0 | 4.7 |
| Cohort 2 | 9 | 3 | 0 | 0 | 0 | 4.8 |
| Cohorts 1 + 2 | 14 | 5 | 0 | 0 | 0 | 4.7 |
| In-person tutor training session | | | | | | |
| Cohort 1 | 2 | 5 | 0 | 0 | 0 | 4.3 |
| Cohort 2 ($n = 10$) | 7 | 1 | 2 | 0 | 0 | 4.5 |
| Cohorts 1 + 2 | 9 | 6 | 2 | 0 | 0 | 4.4 |
| Online tutor curriculum resources (Collaborative Learning Environment page) | | | | | | |
| Cohort 1 | 2 | 3 | 2 | 0 | 0 | 4.0 |
| Cohort 2 | 8 | 4 | 0 | 0 | 0 | 4.7 |
| Cohorts 1 + 2 | 10 | 7 | 2 | 0 | 0 | 4.4 |
| Support from program directors | | | | | | |
| Cohort 1 | 3 | 2 | 2 | 0 | 0 | 4.1 |
| Cohort 2 | 10 | 2 | 0 | 0 | 0 | 4.8 |
| Cohorts 1 + 2 | 13 | 4 | 2 | 0 | 0 | 4.6 |
| Community building with other peer tutors | | | | | | |
| Cohort 1 | 2 | 1 | 3 | 1 | 0 | 3.6 |
| Cohort 2 | 7 | 3 | 2 | 0 | 0 | 4.4 |
| Cohorts 1 + 2 | 9 | 4 | 5 | 1 | 0 | 4.1 |
| Development of learning goals with student | | | | | | |
| Cohort 1 | 3 | 3 | 1 | 0 | 0 | 4.3 |
| Cohort 2 | 7 | 5 | 0 | 0 | 0 | 4.6 |
| Cohorts 1 + 2 | 10 | 8 | 1 | 0 | 0 | 4.5 |
| Clinical skills practice with student (e.g., role-play, standardized patients) | | | | | | |
| Cohort 1 | 3 | 4 | 0 | 0 | 0 | 4.4 |
| Cohort 2 | 7 | 3 | 2 | 0 | 0 | 4.4 |
| Cohorts 1 + 2 | 10 | 7 | 2 | 0 | 0 | 4.4 |

[a]Rated on a 5-point Likert scale (1 = *extremely dissatisfied,* 5 = *extremely satisfied*).

coincided with graduation for some tutors and summer break for others. Survey fatigue may also have played a role. Finally, a majority of tutors attended the focus groups where they were able to provide detailed verbal feedback; they may have felt that they did not have further feedback to share on the survey. Of the students, many started on busy clinical rotations (e.g., third-year clerkships) right after the program; the stress and added cognitive load of the COVID-19 pandemic may also have played a role.

We expanded the program to 18 tutors in the second cohort and continued to find high tutor satisfaction with the in-person training session and tutor curriculum. An added benefit of incorporating the monthly community-building sessions is being able to identify new, evolving tutor needs and iteratively revise the curriculum rapidly. However, we must remain vigilant in ensuring that the quality of longitudinal support and tutor experience is not compromised when growing the program to a larger size. With the smaller first cohort of student participants, each tutor was paired with a single student, which maximized the individual attention and program support that the student and tutor received. Our second cohort had multiple tutors working with more than one student simultaneously, which demonstrated that the tutor curriculum and program design continued to provide sufficient support for tutors and student participants alike.

The program was designed with the goal of implementing a curriculum that improved the knowledge, skills, and confidence of tutors to be successful clinical skills teachers. When we introduced the curriculum to our first tutor cohort, we sought to ensure that the program demonstrated utility and potential benefits that supported future growth and sustainability. We achieved this by assessing usefulness and satisfaction related to curricular materials. We were reassured by the survey and interview data obtained from two cohorts that the curriculum was satisfactory to tutor and student needs. Our future direction is to specifically assess the development of teaching skills and confidence in mastering educational objectives using a combination of tutor self-assessments and students' evaluations of their tutors. We will also conduct semistructured

Copyright © 2022 Chang et al. This is an open-access publication distributed under the terms of the Creative Commons Attribution license.

**Table 3.** Student Participant Ratings of Peer Tutoring Program (Cohort 1 $n = 5$, Cohort 2 $n = 11$)

| Question[a] | No. of Respondents | | | | | Average Rating |
|---|---|---|---|---|---|---|
| | Extremely Satisfied | Somewhat Satisfied | Neutral | Somewhat Dissatisfied | Extremely Dissatisfied | |
| Overall program experience | | | | | | |
| Cohort 1 | 4 | 1 | 0 | 0 | 0 | 4.8 |
| Cohort 2 | 10 | 1 | 0 | 0 | 0 | 4.9 |
| Cohorts 1 + 2 | 14 | 2 | 0 | 0 | 0 | 4.9 |
| Relationship with peer tutor | | | | | | |
| Cohort 1 | 4 | 1 | 0 | 0 | 0 | 4.8 |
| Cohort 2 | 9 | 2 | 0 | 0 | 0 | 4.8 |
| Cohorts 1 + 2 | 13 | 3 | 0 | 0 | 0 | 4.8 |
| Development of learning goals with peer tutor | | | | | | |
| Cohort 1 | 4 | 1 | 0 | 0 | 0 | 4.8 |
| Cohort 2 | 10 | 1 | 0 | 0 | 0 | 4.9 |
| Cohorts 1 + 2 | 14 | 2 | 0 | 0 | 0 | 4.9 |
| Support from program directors | | | | | | |
| Cohort 1 | 4 | 1 | 0 | 0 | 0 | 4.8 |
| Cohort 2 | 8 | 2 | 1 | 0 | 0 | 4.6 |
| Cohorts 1 + 2 | 12 | 3 | 1 | 0 | 0 | 4.7 |
| Effectiveness of peer tutor to provide coaching on clinical skills | | | | | | |
| Cohort 1 | 5 | 0 | 0 | 0 | 0 | 5.0 |
| Cohort 2 | 10 | 1 | 0 | 0 | 0 | 4.9 |
| Cohorts 1 + 2 | 15 | 1 | 0 | 0 | 0 | 4.9 |
| Clinical skills practice with peer tutor (e.g., role-play, standardized patients) | | | | | | |
| Cohort 1 | 4 | 0 | 1 | 0 | 0 | 4.6 |
| Cohort 2 | 7 | 2 | 2 | 0 | 0 | 4.5 |
| Cohorts 1 + 2 | 11 | 2 | 3 | 0 | 0 | 4.5 |

[a]Rated on a 5-point Likert scale (1 = *extremely dissatisfied*, 5 = *extremely satisfied*).

**Table 4.** End-of-Program Focus Group: Tutor Perspectives on the Curriculum

| Key Themes | Illustrative Quotes |
|---|---|
| Strengths | |
| Curriculum structure | "The structure was fantastic with a formal, real curriculum. Also, having the opportunity to use real standardized patients for practice with my student was really fantastic."<br>"Another highlight is the excellent introduction session and the incredibly well-thought-out written materials that accompanied it." |
| Director support | "Having done tutoring for other programs, it was really helpful to have faculty members for support compared to feeling alone in other programs. It was good to touch base in orientation and before working with the student, which made me feel well-supported as a tutor."<br>"I think it was fantastic in having a mentor to bounce ideas off of, especially getting a sense of the student's goals was really helpful, finding what resources I had access to, and how to tackle the student's learning goals. I wish I had taken greater advantage of this resource." |
| Learning resources | "I really appreciate all the links on the CLE webpage to helpful resources. My student has found these helpful and many of my favorite independent learning resources, books, and modules were there. It is impressively well-curated for a first edition CLE page." |
| Areas for improvement | |
| Tutor check-ins; community building; collaboration; information sharing | "My only recommendation is to hold meetings or check-ins with the tutor to brainstorm on how to bring out the best of the student. Every student is different, and I personally felt like I hit a wall with my student on how much we can improve."<br>"I suggest having a check-in towards the middle of the program to get tutors together to discuss how everyone has been running their sessions, which can generate more ideas and help pairs continually improve their sessions." |
| Specific resources | "I think having a concrete list of tips of certain types of practice (e.g., specific and high-yield tips for how to coach students who are struggling with their presentation on the wards) might be a nice addition."<br>"I would like to see more specific resources such as example history and physical exam write-ups, additional standardized or 'real life' cases to use during sessions." |

Abbreviation: CLE, Collaborative Learning Environment.

Copyright © 2022 Chang et al. This is an open-access publication distributed under the terms of the Creative Commons Attribution license.

one-on-one interviews with student learners to receive their feedback on whether tutors were effective in meeting specific tutor milestones. A long-term vision would be to measure a student participant's objective clinical skills performance before and after working with a peer tutor to associate clinical skills success with specific components of our program.

There are multiple ways the CSTP can be expanded or applied to a different learner group. For example, the program could expand to more learners (e.g., beyond remediation context); in doing so, it would be important to consider the bandwidth of the program directors and ensure that the program was still able to provide a high-quality experience for student participants and tutors. Additionally, the program could be expanded to residency or fellowship programs.

In terms of sustainability, it is critical to consider the resources available, particularly in terms of faculty program director time. One innovation that has been successful is recruiting a Lead Tutor. The Lead Tutor is a student participating in the program for a second year (e.g., they participated as a tutor during their gap year and then opted to stay on for their fourth year of medical school). As noted in the Methods section, the Lead Tutor is paid for approximately 5-10 hours per month and can provide essential program support in areas such as tutor payments, arranging monthly tutor community sessions, and providing the tutor perspective while answering questions during recruitment periods. Faculty time for the scope of program described here was approximately 10-20 hours per month in the first 4-6 months and 3-5 hours per month after that. For institutions that choose to use the CSTP curriculum provided, the initial higher effort in the first 4-6 months will likely not be needed as this additional time was dedicated to designing and creating the actual program. For sustainability, it is important to have buy-in from administration and support for tutor payments and other administrative tasks such as room scheduling.

It is also important to consider the local context of an institution when developing and sustaining a program such as the CSTP. For example, our institution has a robust coaching program. Coaches are assigned to two cohorts of approximately six students each in different years; they do clinical skills teaching, systems improvement projects, and clinical reasoning learning in the first 2 years, while providing mentorship and career advising for all 4 years of medical school. In relation to the CSTP, coaches have played important roles including referring students to the program, providing additional context for a student's learning goals, and serving as references for tutors who apply to the program. Implementing this program in a context without such faculty coaches is quite feasible. To do so, it would be important to form close relationships with medical school leaders who know students well (e.g., dean for students, director of clinical skills) and to advertise the program widely (e.g., to clerkship directors and other course directors).

In summary, we designed a tutor curriculum to train peer tutors on teaching skills including building rapport, providing effective feedback, creating learning goals, and guiding students through self-regulated learning principles. The curriculum design resulted in high satisfaction ratings from both tutors and student participants. The inclusion of a formal curriculum may be beneficial in assisting peer tutors with successful navigation of the peer relationship, remediation of the student learner, and maturation of core teaching skills. Our educational objectives and learning materials can help standardize the expectations of a peer tutor while being adapted and implemented by other tutoring programs.

## Appendices

A. Tutor Curriculum Learning Objectives and Content.docx
B. Tutor Curriculum Supplement.docx
C. Tutor Curriculum Nuts and Bolts.docx
D. Tutor Checklist.docx
E. CSTP Facilitator Guide for Tutor Training Session.docx
F. Tutor Training Session Survey.docx
G. Tutor Participant Survey.docx
H. Student Participant Survey.docx

*All appendices are peer reviewed as integral parts of the Original Publication.*

**Chih-Chiun Jamie Chang, MD:** First-Year Resident, Icahn School of Medicine at Mount Sinai; ORCID: https://orcid.org/0000-0002-1728-3372

**Sara-Megumi Rumrill, MD:** Assistant Clinical Professor, Division of VA General Internal Medicine, Department of Medicine, University of California, San Francisco, School of Medicine

**Abigail Phillips, MD:** Associate Clinical Professor, Division of VA General Internal Medicine, Department of Medicine, University of California, San Francisco, School of Medicine

### Disclosures
None to report.

### Funding/Support
None to report.

Copyright © 2022 Chang et al. This is an open-access publication distributed under the terms of the Creative Commons Attribution license.

### Prior Presentations

Chang CC, Phillips A, Rumrill SM. Developing a tutor curriculum for the Clinical Skills Tutoring Program (CSTP) at UCSF. Presented at: Innovations in Medical Education Online Conference; February 18-19, 2021.

Chang CC, Phillips A, Rumrill SM. Developing a tutor curriculum for the Clinical Skills Tutoring Program (CSTP) at UCSF School of Medicine. Poster presented at: Society of General Internal Medicine California-Hawaii and Northwest Virtual Regional Meeting; January 22-23, 2021.

### Ethical Approval

The University of California, San Francisco, Institutional Review Board deemed further review of this project not necessary.

## References


1. Hauer KE, Hodgson CS, Kerr KM, Teherani A, Irby DM. A national study of medical student clinical skills assessment. *Acad Med*. 2005;80(suppl 10):S25-S29. https://doi.org/10.1097/00001888-200510001-00010
2. Ellaway RH, Chou CL, Kalet AL. Situating remediation: accommodating success and failure in medical education systems. *Acad Med.* 2018;93(3):391-398. https://doi.org/10.1097/ACM.0000000000001855
3. Hauer KE, Teherani A, Kerr KM, O'Sullivan PS, Irby DM. Student performance problems in medical school clinical skills assessments. *Acad Med.* 2007;82(suppl 10):S69-S72. https://doi.org/10.1097/ACM.0b013e31814003e8
4. Tamachi S, Giles JA, Dornan T, Hill EJR. "You understand that whole big situation they're in": interpretative phenomenological analysis of peer-assisted learning. *BMC Med Educ*. 2018;18:197. https://doi.org/10.1186/s12909-018-1291-2
5. Field M, Burke JM, McAllister D, Lloyd DM. Peer-assisted learning: a novel approach to clinical skills learning for medical students. *Med Educ*. 2007;41(4):411-418. https://doi.org/10.1111/j.1365-2929.2007.02713.x
6. Hudson JN, Tonkin AL. Clinical skills education: outcomes of relationships between junior medical students, senior peers and simulated patients. *Med Educ*. 2008;42(9):901-908. https://doi.org/10.1111/j.1365-2923.2008.03107.x
7. Burgess A, Dornan T, Clarke AJ, Menezes A, Mellis C. Peer tutoring in a medical school: perceptions of tutors and tutees. *BMC Med Educ*. 2016;16:85. https://doi.org/10.1186/s12909-016-0589-1
8. DeVoe PAH, Couse P, Hess M. Does peer tutoring facilitate medical student learner self-efficacy? [version 1]. *MedEdPublish*. 2016;5:28. https://doi.org/10.15694/mep.2016.000028
9. Akinla O, Hagan P, Atiomo W. A systematic review of the literature describing the outcomes of near-peer mentoring programs for first year medical students. *BMC Med Educ*. 2018;18:98. https://doi.org/10.1186/s12909-018-1195-1
10. Soriano RP, Blatt B, Coplit L, et al. Teaching medical students how to teach: a national survey of students-as-teachers programs in U.S. medical schools. *Acad Med.* 2010;85(11):1725-1731. https://doi.org/10.1097/ACM.0b013e3181f53273
11. Burgess A, McGregor D, Mellis C. Medical students as peer tutors: a systematic review. *BMC Med Educ*. 2014;14:115. https://doi.org/10.1186/1472-6920-14-115
12. Buckley S, Zamora J. Effects of participation in a cross year peer tutoring programme in clinical examination skills on volunteer tutors' skills and attitudes towards teachers and teaching. *BMC Med Educ*. 2007;7:20. https://doi.org/10.1186/1472-6920-7-20
13. Chou CL, Kalet A, Costa MJ, Cleland J, Winston K. Guidelines: the dos, don'ts and don't knows of remediation in medical education. *Perspect Med Educ*. 2019;8(6):322-338. https://doi.org/10.1007/s40037-019-00544-5
14. Harris D. Being an effective tutor. *MedEdPORTAL*. 2006;2:222. https://doi.org/10.15766/mep_2374-8265.222
15. Bell M, Defilippo C, Miloslavsky E, et al. Medical school peer-tutoring training curricula. *MedEdPORTAL*. 2010;6:7810. https://doi.org/10.15766/mep_2374-8265.7810
16. Whitmill A, Edwards T, Charles S. Training medical student facilitators of peer-assisted study sessions using an objective standardized teaching exercise. *MedEdPORTAL*. 2020;16:10898. https://doi.org/10.15766/mep_2374-8265.10898
17. Lockspeiser TM, O'Sullivan P, Teherani A, Muller J. Understanding the experience of being taught by peers: the value of social and cognitive congruence. *Adv Health Sci Educ Theory Pract.* 2008;13(3):361-372. https://doi.org/10.1007/s10459-006-9049-8
18. Leggett H, Sandars J, Roberts T. Twelve tips on how to provide self-regulated learning (SRL) enhanced feedback on clinical performance. *Med Teach.* 2019;41(2):147-151. https://doi.org/10.1080/0142159X.2017.1407868
19. Cutrer WB, Miller B, Pusic MV, et al. Fostering the development of Master Adaptive Learners: a conceptual model to guide skill acquisition in medical education. *Acad Med*. 2017;92(1):70-75. https://doi.org/10.1097/ACM.0000000000001323
20. Sandars J, Cleary TJ. Self-regulation theory: applications to medical education: AMEE Guide no. 58. *Med Teach*. 2011;33(11): 875-886. https://doi.org/10.3109/0142159X.2011.595434


**Received:** July 11, 2021
**Accepted:** December 1, 2021
**Published:** February 14, 2022

Copyright © 2022 Chang et al. This is an open-access publication distributed under the terms of the Creative Commons Attribution license.
