# Supplementary figures and images for: Clinical Skills Tutoring Program (CSTP): Developing a Curriculum for Medical Student Clinical Skills Peer Tutors
Source: MedEdPORTAL. 2022 Feb 14;18:11225. doi: 10.15766/mep_2374-8265.11225 (PMC8841391; doi:10.15766/mep_2374-8265.11225)

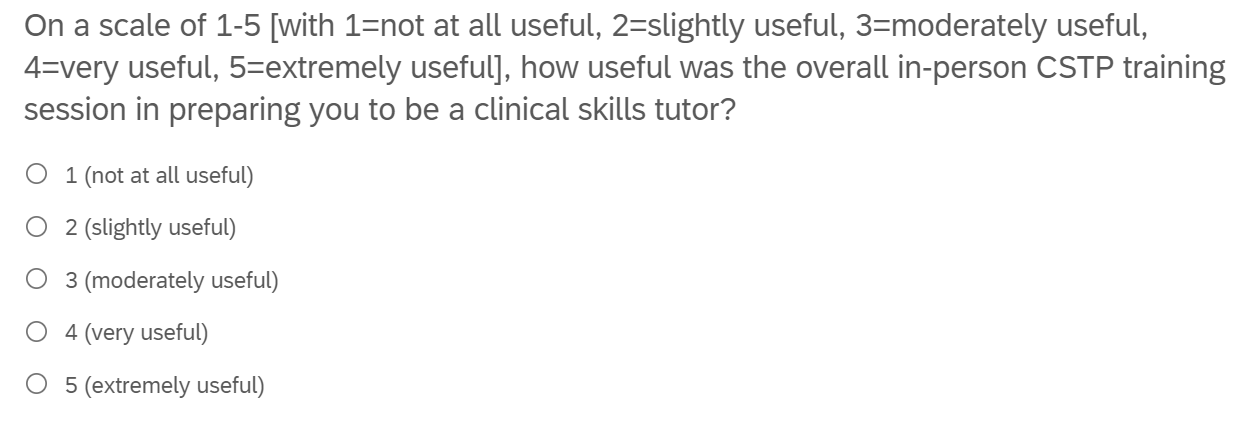

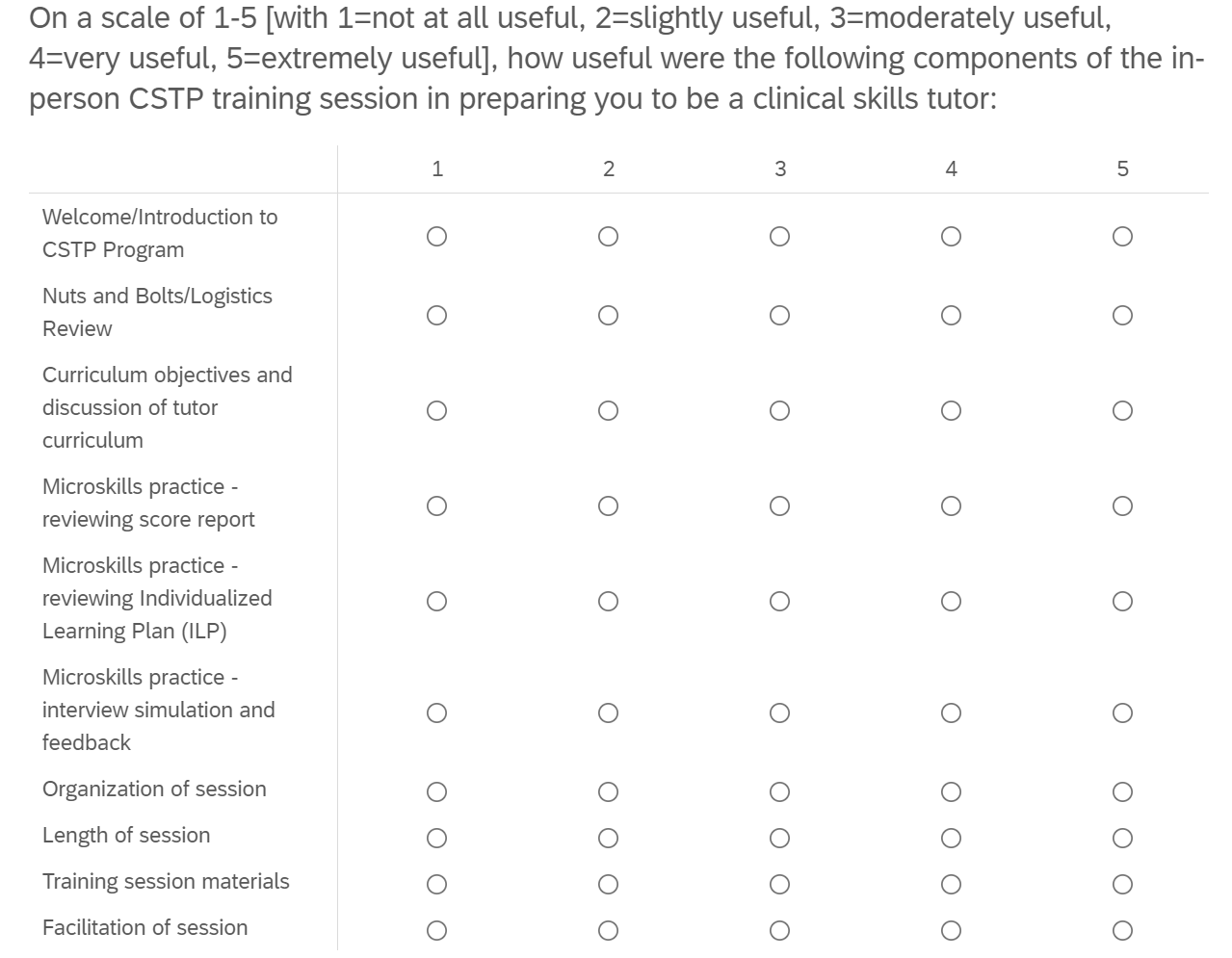

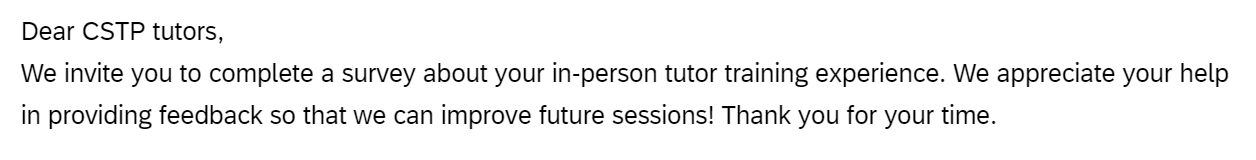


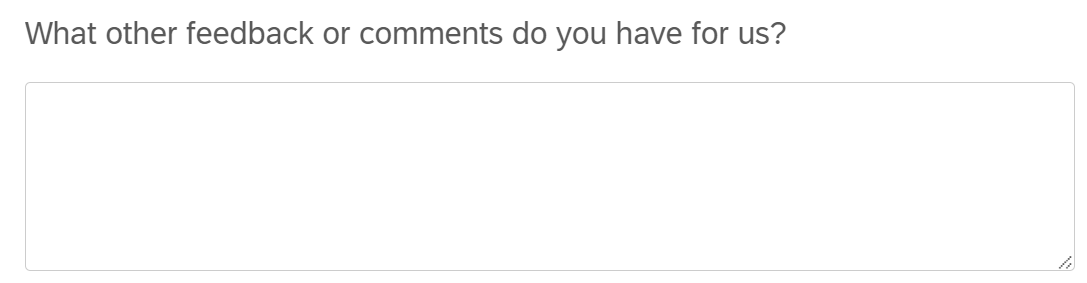

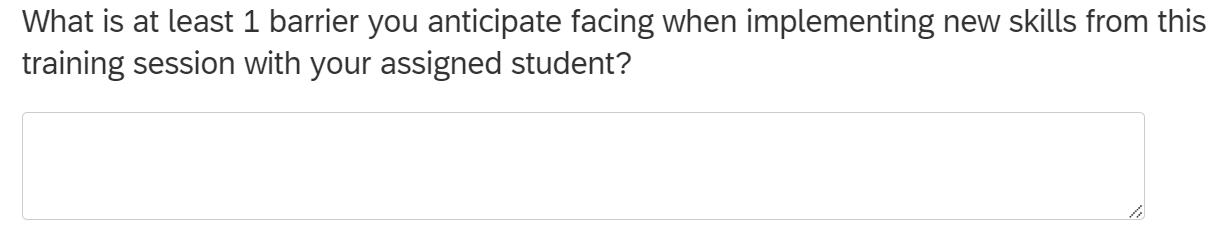

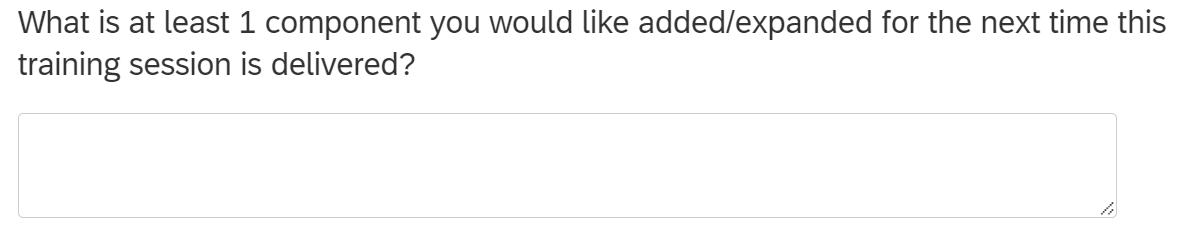

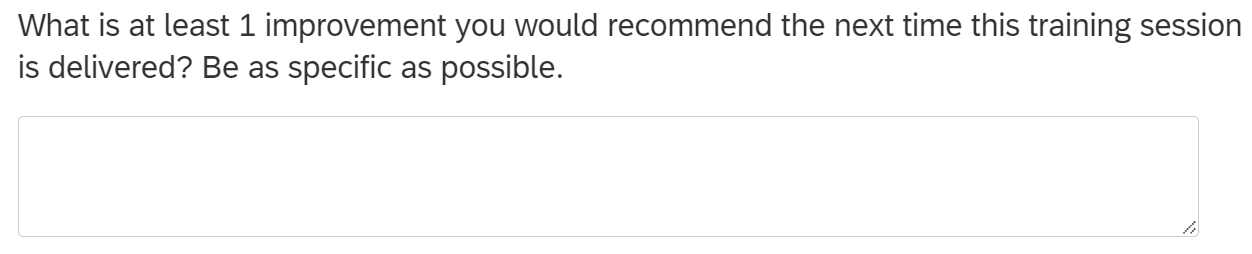

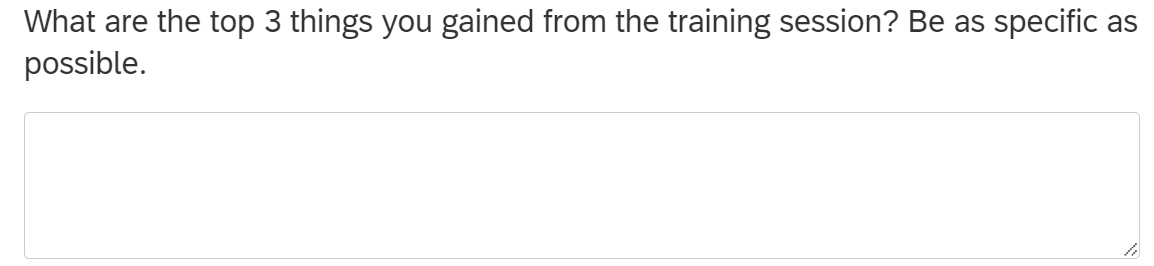

Supplement: Supplementary file 1 — Tutor Curriculum Learning Objectives and Content.docxTutor Curriculum Supplement.docxTutor Curriculum Nuts and Bolts.docxTutor Checklist.docxCSTP Facilitator Guide for Tutor Training Session.docxTutor Training Session Survey.docxTutor Participant Survey.docxStudent Participant Survey.docx [file mep_2374-8265.11225-s001.zip › F. Tutor Training Session Survey.docx]

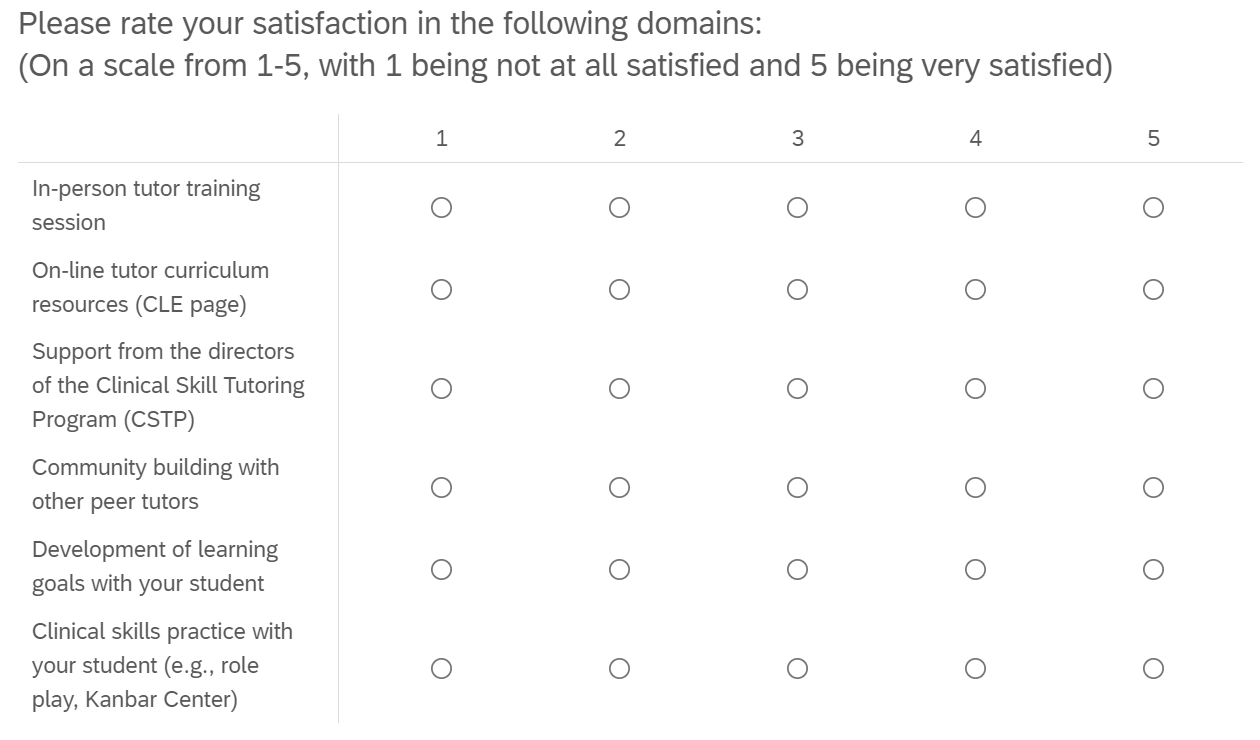

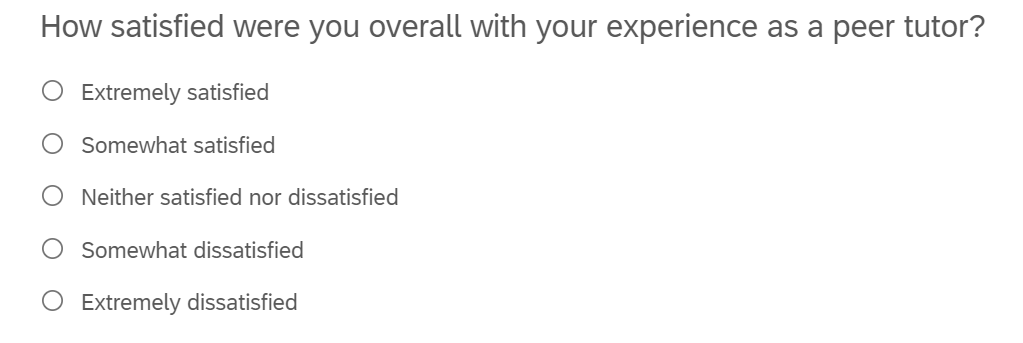

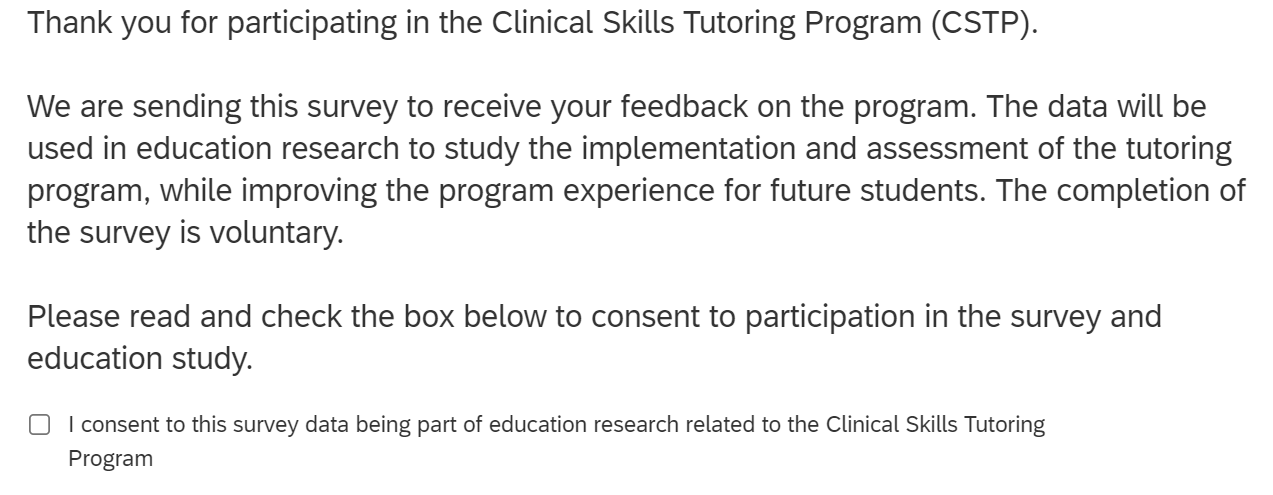


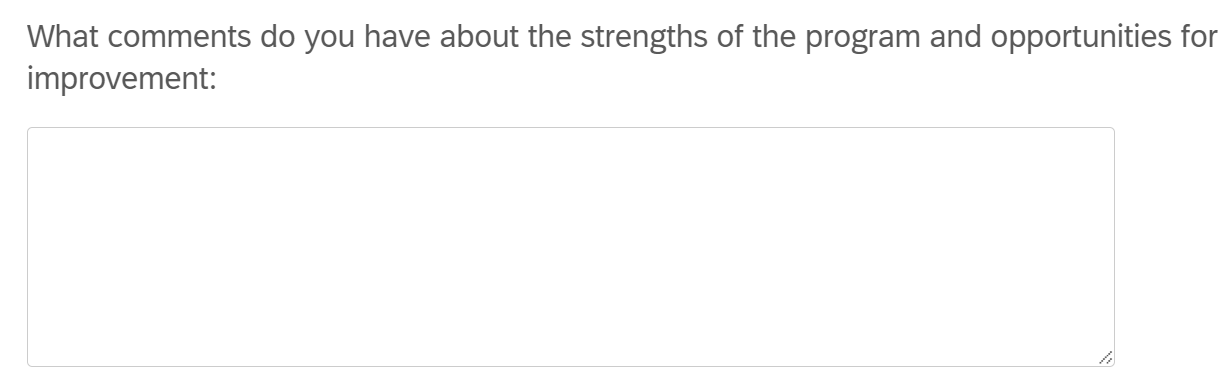

Supplement: Supplementary file 1 — Tutor Curriculum Learning Objectives and Content.docxTutor Curriculum Supplement.docxTutor Curriculum Nuts and Bolts.docxTutor Checklist.docxCSTP Facilitator Guide for Tutor Training Session.docxTutor Training Session Survey.docxTutor Participant Survey.docxStudent Participant Survey.docx [file mep_2374-8265.11225-s001.zip › G. Tutor Participant Survey.docx]

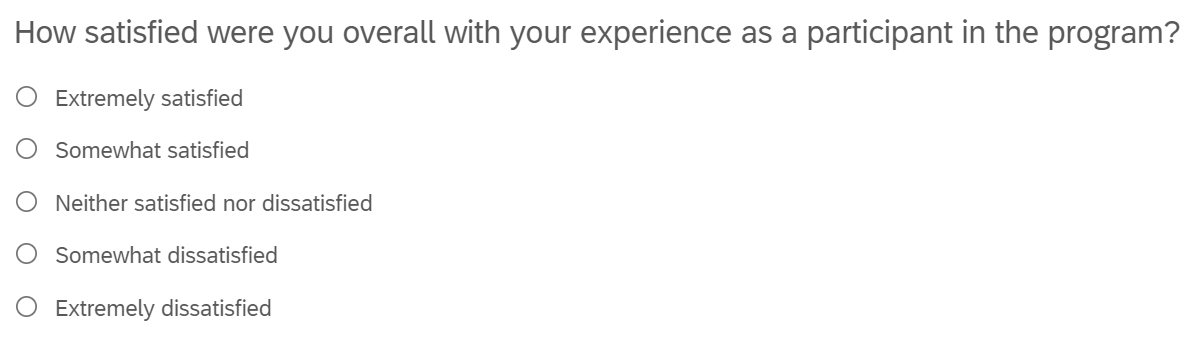

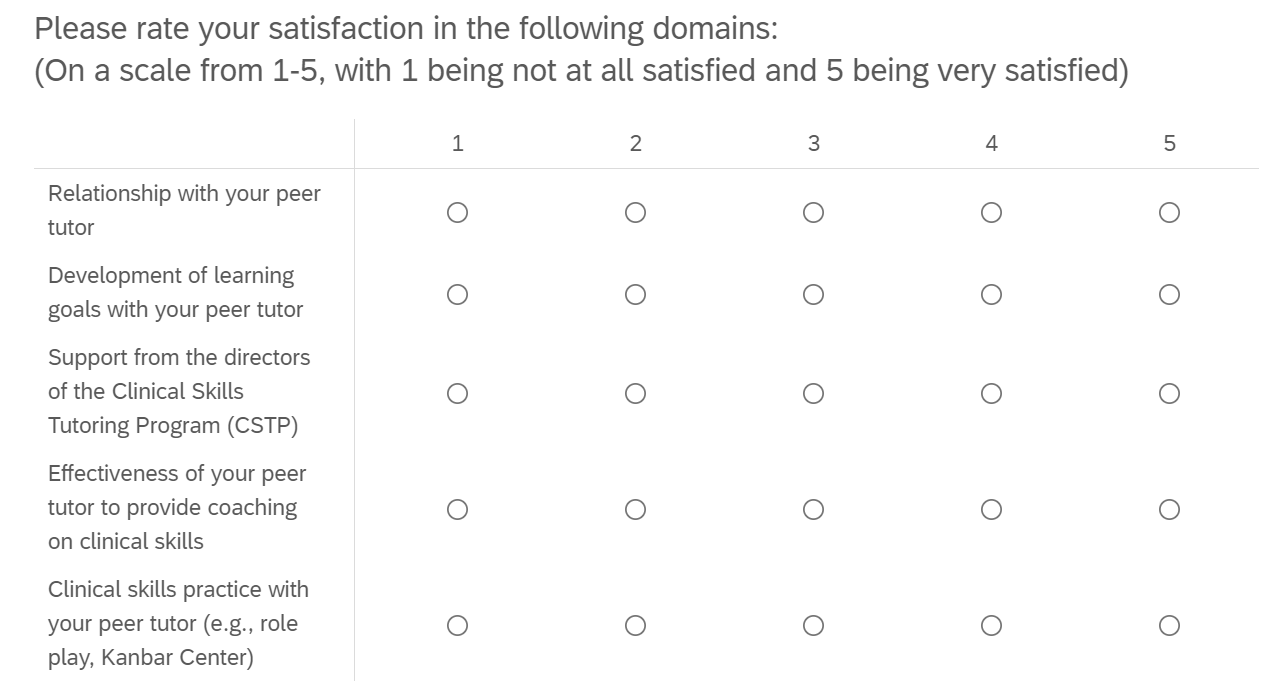

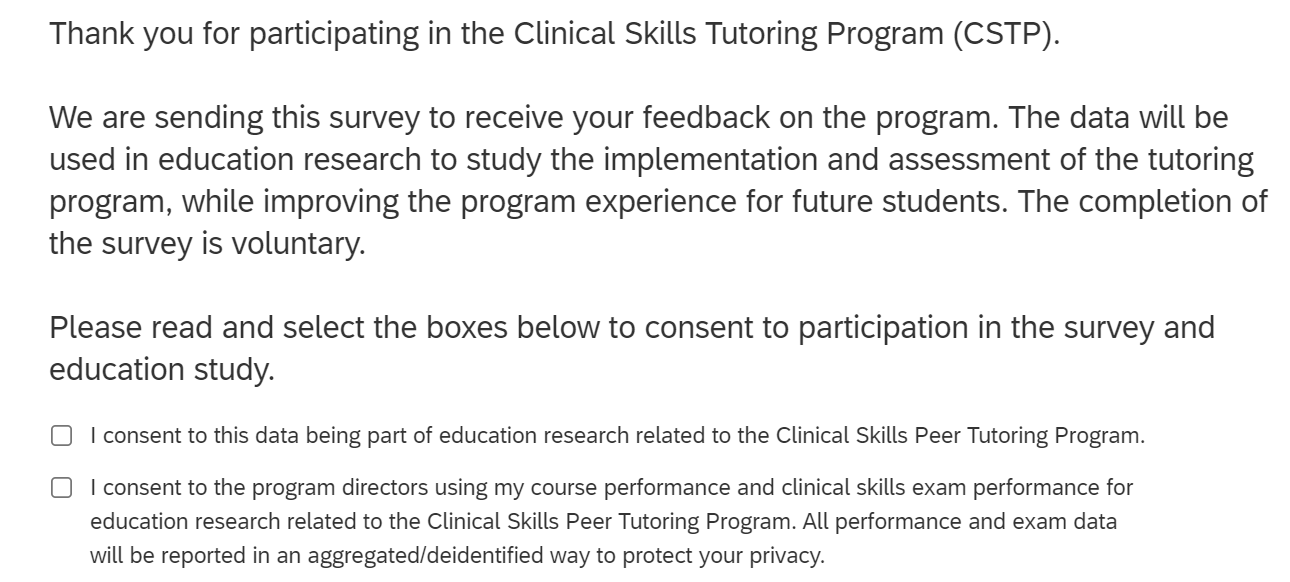


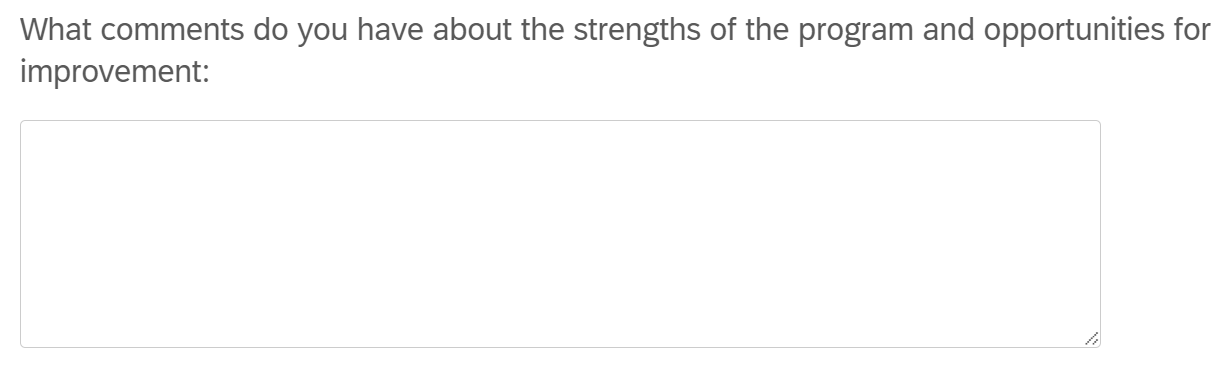

Supplement: Supplementary file 1 — Tutor Curriculum Learning Objectives and Content.docxTutor Curriculum Supplement.docxTutor Curriculum Nuts and Bolts.docxTutor Checklist.docxCSTP Facilitator Guide for Tutor Training Session.docxTutor Training Session Survey.docxTutor Participant Survey.docxStudent Participant Survey.docx [file mep_2374-8265.11225-s001.zip › H. Student Participant Survey.docx]
